# A survey of Sub-Saharan African medical schools

**DOI:** 10.1186/1478-4491-10-4

**Published:** 2012-02-24

**Authors:** Candice Chen, Eric Buch, Travis Wassermann, Seble Frehywot, Fitzhugh Mullan, Francis Omaswa, S Ryan Greysen, Joseph C Kolars, Delanyo Dovlo, Diaa Eldin El Gali Abu Bakr, Abraham Haileamlak, Abdel Karim Koumare, Emiola Oluwabunmi Olapade-Olaopa

**Affiliations:** 1 Department of Health Policy, The George Washington University, Washington, DC, USA; 2 School of Health Systems and Public Health, University of Pretoria, Pretoria, South Africa; 3 African Centre for Global Health and Social Transformation, Kampala, Uganda; 4 Robert Wood Johnson Clinical Scholar, Yale University School of Medicine, New Haven, CT, USA; 5 Department of Internal Medicine, University of Michigan, Ann Arbor, MI, USA; 6 Ministry of Health, Accra, Ghana; 7 Department of Mental Health, University of Gezira, Wad Medani, Sudan; 8 Department of Pediatrics and Child Health, Jimma University, Jimma, Ethiopia; 9 Department of Anatomy and Surgery, University of Mali, Bamako, Mali; 10 Department of Surgery, University of Ibadan, Ibadan, Nigeria

## Abstract

**Background:**

Sub-Saharan Africa suffers a disproportionate share of the world's burden of disease while having some of the world's greatest health care workforce shortages. Doctors are an important component of any high functioning health care system. However, efforts to strengthen the doctor workforce in the region have been limited by a small number of medical schools with limited enrolments, international migration of graduates, poor geographic distribution of doctors, and insufficient data on medical schools. The goal of the Sub-Saharan African Medical Schools Study (SAMSS) is to increase the level of understanding and expand the baseline data on medical schools in the region.

**Methods:**

The SAMSS survey is a descriptive survey study of Sub-Saharan African medical schools. The survey instrument included quantitative and qualitative questions focused on institutional characteristics, student profiles, curricula, post-graduate medical education, teaching staff, resources, barriers to capacity expansion, educational innovations, and external relationships with government and non-governmental organizations. Surveys were sent via e-mail to medical school deans or officials designated by the dean. Analysis is both descriptive and multivariable.

**Results:**

Surveys were distributed to 146 medical schools in 40 of 48 Sub-Saharan African countries. One hundred and five responses were received (72% response rate). An additional 23 schools were identified after the close of the survey period. Fifty-eight respondents have been founded since 1990, including 22 private schools. Enrolments for medical schools range from 2 to 1800 and graduates range from 4 to 384. Seventy-three percent of respondents (n = 64) increased first year enrolments in the past five years. On average, 26% of respondents' graduates were reported to migrate out of the country within five years of graduation (n = 68). The most significant reported barriers to increasing the number of graduates, and improving quality, related to infrastructure and faculty limitations, respectively. Significant correlations were seen between schools implementing increased faculty salaries and bonuses, and lower percentage loss of faculty over the previous five years (*P *= 0.018); strengthened institutional research tools (*P *= 0.00015) and funded faculty research time (*P *= 0.045) and greater faculty involvement in research; and country compulsory service requirements (*P *= 0.039), a moderate number (1-5) of post-graduate medical education programs (*P *= 0.016) and francophone schools (*P *= 0.016) and greater rural general practice after graduation.

**Conclusions:**

The results of the SAMSS survey increases the level of data and understanding of medical schools in Sub-Saharan Africa. This data serves as a baseline for future research, policies and investment in the health care workforce in the region which will be necessary for improving health.

## Background

Sub-Saharan Africa suffers a disproportionate share of the world's burden of disease while also struggling under some of the greatest health care workforce shortages. Twelve per cent of the world's population lives in Sub-Saharan Africa [1], yet the region suffers 27% of the world's total burden of disease, has only 3.5% of the world's health care workforce and 1.7% of the world's physicians [2]. A stable and sufficient health care workforce is essential to meet the health care needs of any population. Increasing recognition of this fact and of the critical need in Africa has become the recent focus of global attention [3-6].

While doctors are only one component of any health care workforce, a strong core of medical doctors is necessary in any system to provide high level clinical care and research, and to participate as health care leaders and educators. Sub-Saharan Africa has an estimated 145 000 physicians or 18 physicians per 100 000 people [7]. Some countries, such as the United Republic of Tanzania and Malawi, report as few as 2 physicians per 100 000 people. No other region of the world faces comparable physician shortages: physician numbers in other World Health Organization regions range from 49 to 318 physicians per 100 000 people [8].

Medical schools are the primary institutions that train and graduate medical doctors. They are also integral partners in the training of other health care workers, such as nurses, dentists, pharmacists, and health officers. A significant limiting factor in health care workforce expansion in the past has been small medical school outputs--both due to a low overall number of medical schools and low enrolments at each school [9]. The tendency of graduates to migrate out of the country or to locate in urban areas has also negatively impacted the ability of medical schools to contribute to an optimal health care workforce [10]. Efforts to expand the health care workforce have been further limited by insufficient understanding and data on medical education institutions [11].

The Sub-Saharan African Medical School Study (SAMSS) was designed to establish a baseline understanding of medical schools in Sub-Saharan Africa to inform future policies, plans and investments. For the purposes of this study, Sub-Saharan Africa was considered to include the island nations of Cape Verde, Comoros, Madagascar, Mauritius, Sao Tome & Principe, and Seychelles as well as all of continental Africa except northern Africa (Western Sahara, Morocco, Libya, Tunisia, Algeria, and Egypt). SAMSS included four primary components: a comprehensive literature review, a series of key informant interviews, site visits to 10 medical schools, and a survey of all identified Sub-Saharan African medical schools. SAMSS was conducted as a multinational, multi-institutional collaboration of medical educators, researchers and policy makers acting through three bodies. These were the Secretariat based at the George Washington University; the Advisory Committee made up of representatives of the ten African site visited medical schools and six African policy leaders serving as at-large members; and the University of Pretoria School of Health Systems and Public Health chosen on a competitive basis to assist in the design and implementation of the SAMSS survey. The overall findings of SAMSS, including recommendations, have been published in the peer-reviewed literature [12] and in a limited print study culmination report [13]. However, the survey results published were a subset of the overall survey findings and the full extent of information about African medical education collected in the SAMSS survey has not been published. This article describes the full findings of the survey of all medical schools, providing a level of detail and access to the data that will be needed to guide future programs and evaluations.

## Methods

The SAMSS survey is a descriptive survey of Sub-Saharan African medical schools. The survey instrument (Additional File 1) was developed based upon previous surveys of medical and health professional schools in Africa and globally [14-17], key informant interviews, and input from the SAMSS Advisory Committee. The survey instrument included quantitative and qualitative questions focused on institutional characteristics, student profiles, curricula, post-graduate medical education, teaching staff, resources, barriers to quality and capacity expansion, educational innovations, and external relationships with government and nongovernmental organizations. Questions were multiple choice, short answers, and open-ended. Adequacy of resources and barriers to increasing the quality and quantity of graduates were rated on 0-4 Likert scales (resource: does not exist - good; barrier: not a barrier - severe). The survey instrument was pilot tested with nine SAMSS Advisory Committee members, all either deans or high-level faculty within SSA medical schools.

A list of Sub-Saharan African medical schools (Additional File 2) was compiled using publicly available global directories of medical schools, contact lists of medical education meetings hosted by the World Health Organization in 2002 and the Conférence Internationale des Doyens et des Facultés de Médecine d'Expression Française (CIDMEF) in 2009, and the database of the Global Health Workforce Alliance. University websites were searched, national medical professional registration bodies and Ministries of Health were contacted, and international telephone directory inquiries were used. SAMSS Advisory Committee members and other medical education experts reviewed the compiled list for accuracy and comprehensiveness. In addition, one survey question was designed to elicit the total number of medical schools in a country. This number was checked against the study's working list of medical schools. The process of updating the medical schools list continued through the entire data collection period.

**Figure 1:**
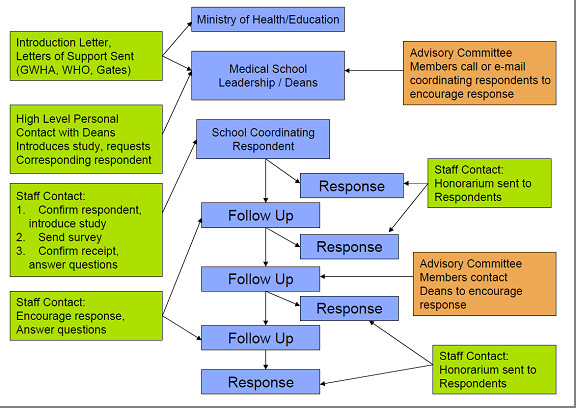
**SAMSS survey plan**.

Initial contact to explain the study and request their support was made with medical school deans through a brief introductory e-mail and/or telephone call. The survey instrument materials (survey, informed consent document, and letters of support from the Gates Foundation, WHO, and GHWA) were then e-mailed to the dean or an appropriate respondent identified by the dean. Periodic follow up was conducted through e-mail and/or telephone calls (Figure 1). In some cases, SAMSS Advisory Committee members encouraged responses through individual contact. Respondents were also offered a US$ 150 honorarium for completing and returning the survey. Surveys were translated into French and Portuguese and sent in the respondent's language of choice.

The study protocol, survey, and supporting information received ethical approval from the Committee for Research on Human Subjects of the Faculty of Health Science of the University of Pretoria (Approval number: 20/2009). All respondents were fully informed about the objectives and content of the study and the potential risks and benefits, and consented to participate. There were no risks to subjects or schools for non-participation. Confidentiality was maintained by reporting findings primarily in aggregates. Only basic identifiable profile information of schools was made publicly available and all respondents were informed of this prior to participation.

Questionnaires were distributed and collected in Microsoft Word 2003 format. Data were double entered into STATA/IC 11.0 and cleaned and analysed using STATA. The major analysis was descriptive in nature, though issues of particular interest were examined by multivariable analysis. Responses to open ended questions were coded then organized into categories.

Various types of multivariable analysis were used, as appropriate. For analysis of correlates of resources, respondents' Likert-scale evaluations of various resources were combined by simple summation into resource indices. These summary indices were used as outcome variables in a complete case analysis of covariance (ANCOVA) or multiple linear regression, as appropriate. Independent variables tested included the age of the school, the school's ownership (public or private), use of each of the four main languages as modes of instruction (English, French, Portuguese, and Arabic), tuition charged per year, size of the entering medical school class (quartile), region of the continent, percent of faculty involved in research (quartile), national gross domestic product (GDP) per capita (PPP, log scale), and population of the country. Variances of country-level independent variables (such as GDP per capita) were clustered at the country level. Independent variables that were not statistically significant predictors were eliminated one by one until all remaining variables were statistically significant at a 95% confidence level. Each eliminated independent variable was individually added back into the model once at that point to retest for statistical significance. Additional outcome variables of interest were examined with similar methods, testing additional independent variables as appropriate. Correlations of pairs of ordinal variables were examined using Pearson's Chi Squared test.

## Results

### Core characteristics

**Figure 2:**
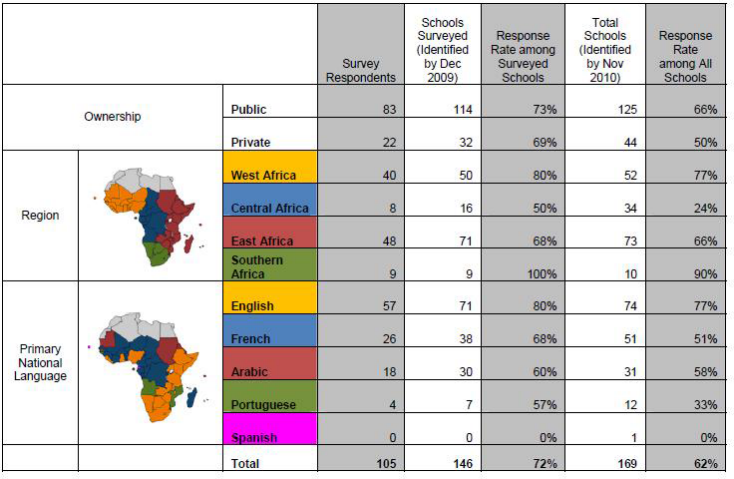
**SAMSS survey respondent demographics**.

Surveys were distributed to 146 medical schools in 40 of 48 Sub-Saharan African countries. One hundred and five schools returned surveys, a response rate of 72%. Response was greatest in English speaking countries. The lowest response rate was seen in Central Africa. Public and private schools responded at similar rates (Figure 2).

An additional 23 schools were identified after the close of the survey period. Six of these schools began admitting medical students in 2009. Of the 17 late identified schools that were open at the start of SAMSS, 13 were private schools in the Democratic Republic of Congo. The survey response rate accounting for all identified SSA medical schools is 62%, with the lowest response (24%) in Central Africa.

The following results represent only those 105 schools that returned surveys. As some respondents did not answer some questions, the number of responses is reported for each result and the percent is calculated based on this number.

**Figure 3:**
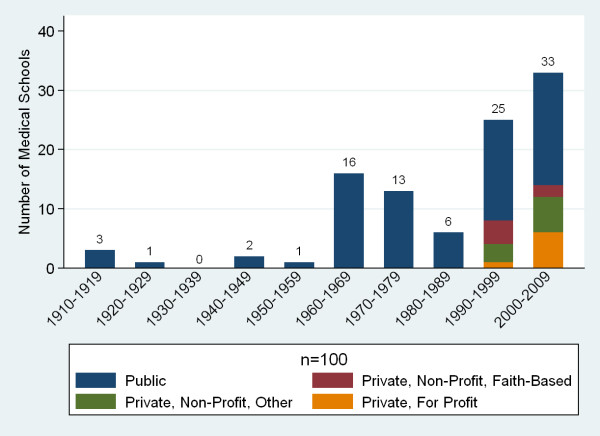
**Date of establishment of sub-Saharan African medical schools by ownership**.

**Figure 4:**
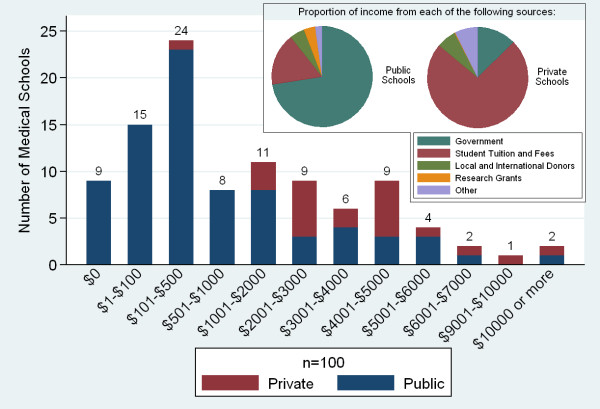
**Sub-Saharan African medical school tuition and sources of income**.

Medical education in SSA began as early as 1918 at Cheikh Anta Diop University (then known as the Ecole Africaine de Médecine et de Pharmacie de Dakar) in Senegal. However, growth in the number of medical schools was slow until the years of independence in Africa in the 1960s and 1970s. The past two decades have seen a particularly steep increase in the number of new medical schools (58 respondents) that included 22 new private medical schools (Figure 3). Responding private medical schools included 6 faith-based not-for-profit schools, 9 non-faith-based not-for-profit schools, and 7 private for-profit schools. Tuition fees in schools ranged from US$0 to US$14, 000. Private school incomes were generally more heavily dependent on tuition fees than public schools (Figure 4). The largest portion of reported medical school expenditures was for professional personnel, on average 45% of expenditures (n = 95).

**Figure 5:**
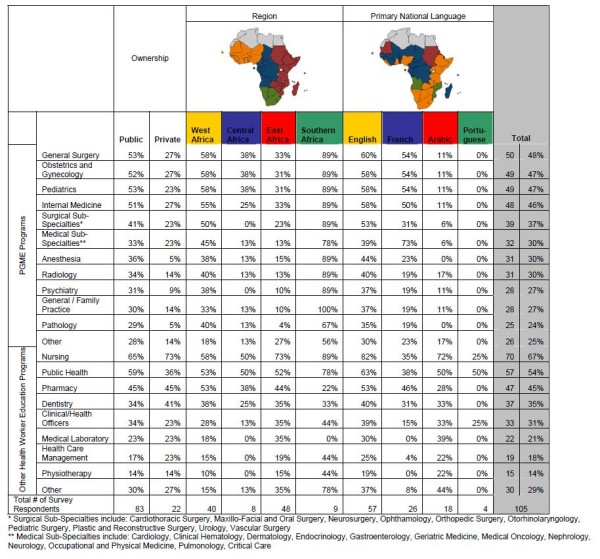
**Sub-Saharan African medical schools post-graduate medical education and other health care workers training programs**.

A majority of responding medical schools reported an affiliation with a university (98%, n = 105) and many schools trained other categories of health care workers, most commonly nurses and public health practitioners, and offered post-graduate medical education (Figure 5).

**Figure 6:**
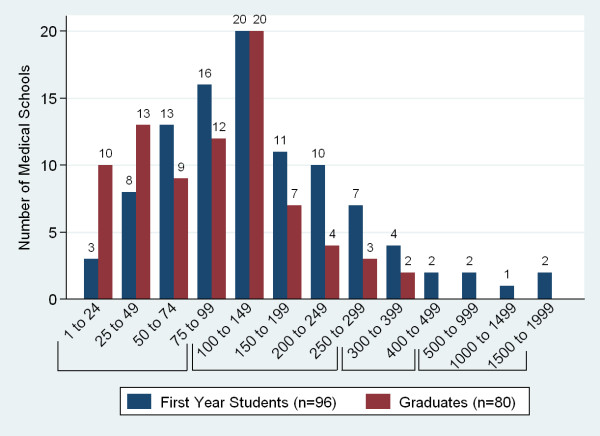
**Sub-Saharan African medical schools first year enrolments and graduates**.

Responding schools reported first year enrolments ranging from 2 to 1800 and graduate numbers from 4 to 384 (Figure 6). The number of applicants varied widely with nearly half of respondents (49%, n = 81) receiving over 500 applications and 32% receiving fewer than 200 applications. A high proportion of those enrolled graduate (mean = 81%, SD = 21.0). Schools reported the largest proportion of their student attrition coming from academic failure (77%, n = 79).

**Figure 7:**
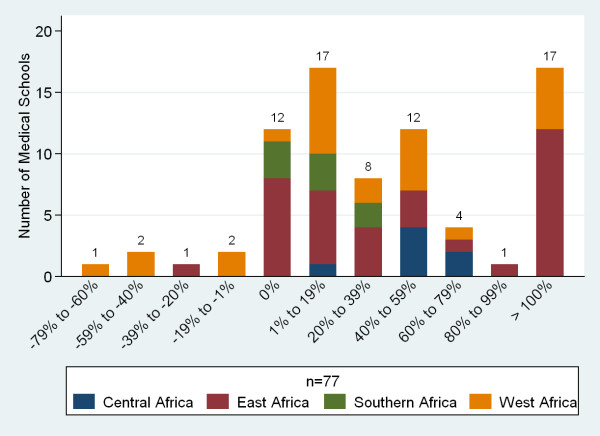
**Percentage change in first year enrolment over the past five years**.

**Figure 8:**
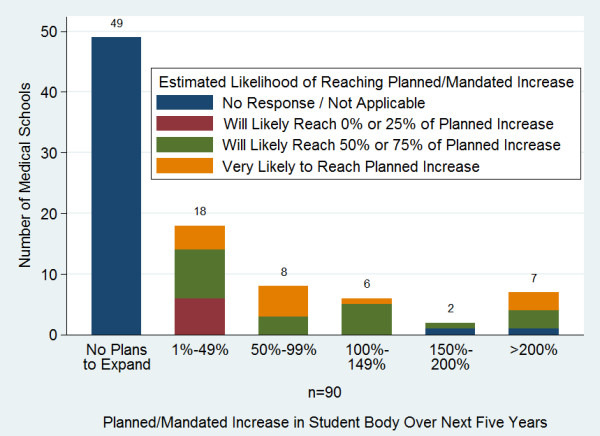
**Planned percentage increase in enrolment over the next five years and likelihood of reaching enrolment goals**.

Seventy-three per cent (n = 64) of respondents reported increases in their first year enrolments in the past five years. Nineteen per cent more than doubled their enrolment (Figure 7). An additional 40 schools (45%, n = 89) reported plans to increase enrolment over the next five years, however, only 36% (n = 45) of respondents felt they were likely to reach their goal increase while 56% indicated they were likely to increase enrolment but not reach their goal (Figure 8). Fifty-eight percent of respondents (n = 96) had received mandates to increase enrolment, most often from ministries of education (44%) or ministries of health (31%).

**Figure 9:**
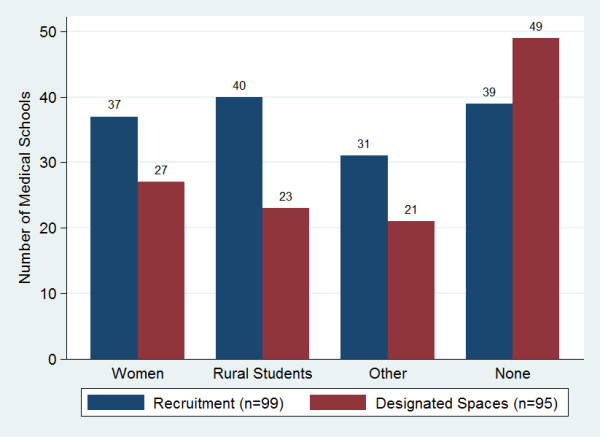
**Sub-Saharan African medical schools focused on student recruitment and reserved positions**.

Many schools reported focused recruitment to increase class diversity and reserved spaces to encourage application and enrolment from specific groups, most often women and rural students (Figure 9). Thirty-eight percent of respondents (n = 101) offered student preparatory programs--defined as any program offered prior to medical school entry to specifically prepare students for the medical school curriculum and improve performance during the medical school years. Forty-five percent of these schools required all students to participate in the preparatory program.

**Figure 10:**
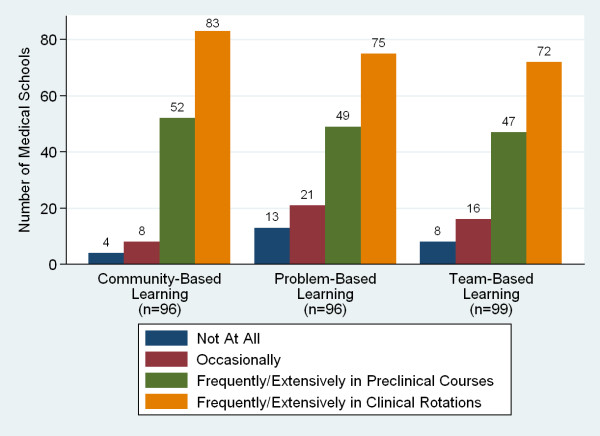
**Sub-Saharan African medical schools use of learning approaches**.

Schools generally required 5 to 7 years for students to graduate (96%, n = 103). Forty-six percent required 6 years. Responding schools reported frequent use of community-based learning, multi-disciplinary team-based learning, and problem-based learning (Figure 10). Fifty-two percent of respondents (n = 101) reported using the internet to augment teaching, eight percent used video distance learning and 22% used online curricula. A majority of schools also required undergraduate students to engage in research activities in order to graduate. Eighty-one percent (n = 100) required a research report or thesis to graduate.

**Figure 11:**
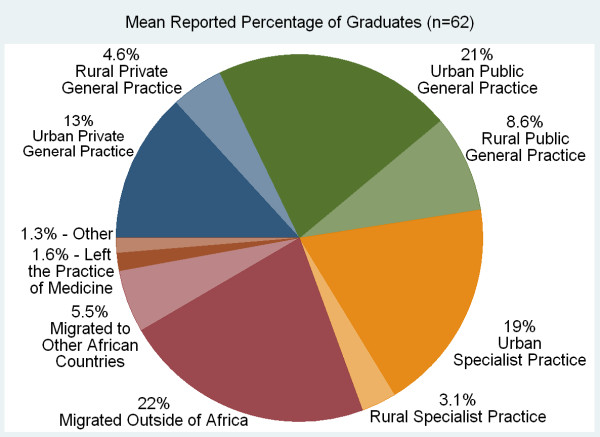
**Mean Estimated location of sub-Saharan African medical school graduates (%) five years after graduation**.

**Figure 12:**
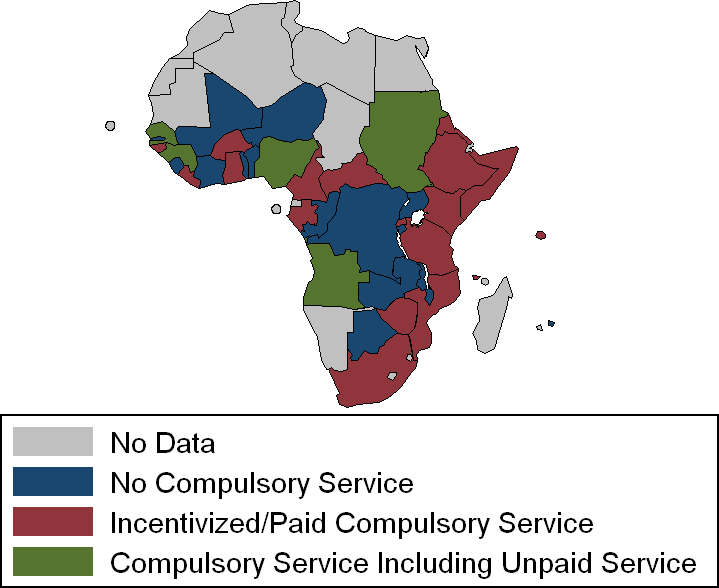
**Sub-Saharan Africa compulsory service requirements by country reported by medical schools**.

Responding schools reported high graduate migration rates. On average, 26% of responding schools' (n = 68) domestic graduates were reported to have migrated out of their country within five years after graduation, with 80% of that emigration being to countries outside of Africa (Figure 11). Five years after graduation an average of 53% of graduates were reported to practice in urban settings in the country compared to 13% in rural settings. Few schools reported graduate tracking systems (18%, n = 67). Location of graduates was most often based on respondent estimates (69%). Sixty-nine per cent of respondents (n = 103) in 21 of the 35 countries with responding schools reported compulsory service requirements for graduates (Figure 12). Of note, in eight countries with more than one school responding to the survey, respondents provided inconsistent answers regarding the compulsory service requirements. Answers were consistent in eight countries and in 20 countries there was only one responding school.

**Figure 13:**
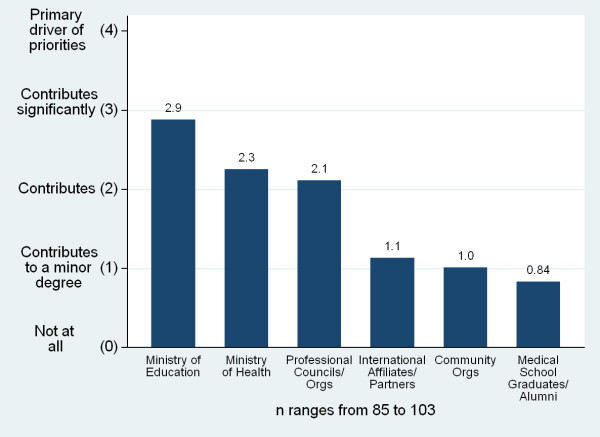
**Participation of external organizations in setting sub-Saharan African medical school priorities**.

Medical schools reported a number of relationships with external organizations. Ministries of Education and Ministries of Health were most frequently reported as significant drivers of medical school priorities (Figure 13). Sixty-three per cent (n = 102) of respondents reported that their national government or professional councils set competencies for medical doctors in their country. An additional 21% reported a list of expected tasks or skills for doctors. Fifty-nine per cent of respondents (n = 85) reported measurement tools for all of the competencies or tasks/skills, 15% reported tools for some, and 26% reported no measurement tools but felt faculty had a general idea of competencies. Many schools also reported that they participated in setting their country's health strategies and policies, through participation on councils or committees (42%, n = 103), informally advising the government (37%), generating research to inform policies (31%), and submitting written recommendations (28%).

Eighty-one percent of responding schools (n = 104) reported undergoing accreditation or formal evaluation through an external organization, most often by national level medical councils or university commissions. Some schools reported accreditation by international organizations, most notably a number of the francophone responding schools reported accreditation by the Conférence Internationale des Doyens et des Facultés de Médecine d'Expression Française (CIDMEF). While some countries with more than one responding school consistently reported accreditation (Ghana, Nigeria, Senegal, South Africa and Tanzania), other countries' schools reported accreditation by different bodies or no accreditation at all.

**Figure 14:**
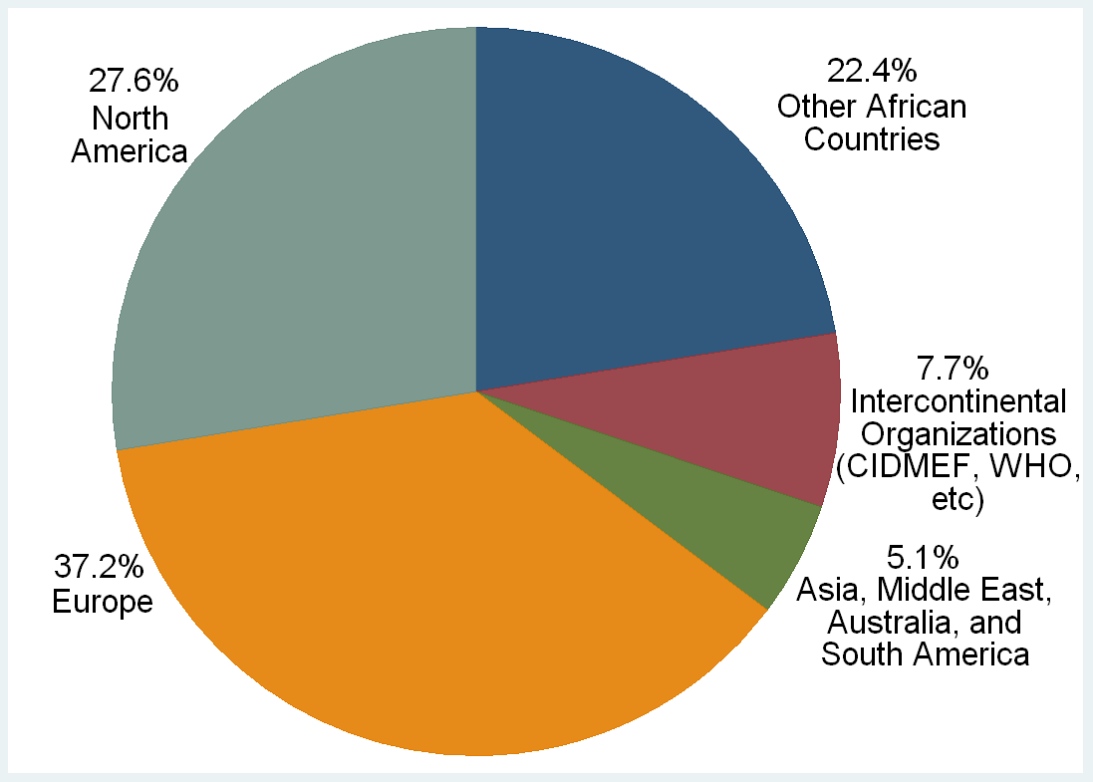
**Sub-Saharan African medical schools - international collaborations**.

Medical schools reported a number of international collaborations, most often with European and North American institutions (Figure 14). Collaborations were most often reported in research, curriculum review, internship programs, distance learning, student and faculty exchanges, and capacity building of faculty.

### Resource capacity

The SAMSS survey examined the personnel and physical resource capacity of Sub-Saharan African medical schools as well as the perceived barriers for graduate scale-up. The total number of teaching staff at medical schools ranged from 1 to 2023. Faculty members were predominantly male and country nationals. The teaching staff was primarily paid by the medical school (69%, n = 98) followed by the teaching hospital (11%). Few faculty members were involved in grant-supported research. More faculty members supplemented their salaries through private practice (Table 1).

**Table 1:** Sub-Saharan African medical school's faculty demographics

	Mean	Standard deviation	n (schools answering applicable question)	25^th^ percentile	50^th^ percentile	75^th^ percentile
Total number of teaching staff	153	257	98	49	90	147

% of Faculty who are female	24%	18%	94	10%	20%	30%

% of Faculty who are foreign	11%	18%	78	1%	3%	10%

% of Faculty in grant-supported research	13%	19%	82	1%	5%	15%

% of Faculty supplementing salary w/private practice	45%	34%	70	12%	44%	75%

**Figure 15:**
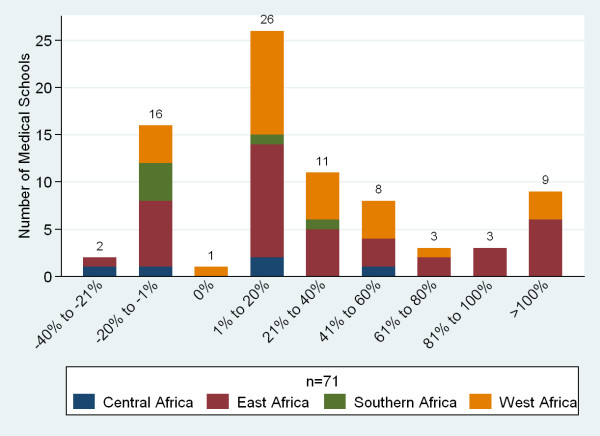
**Percentage change in sub-Saharan African medical school's faculty over the past five years**.

**Figure 16:**
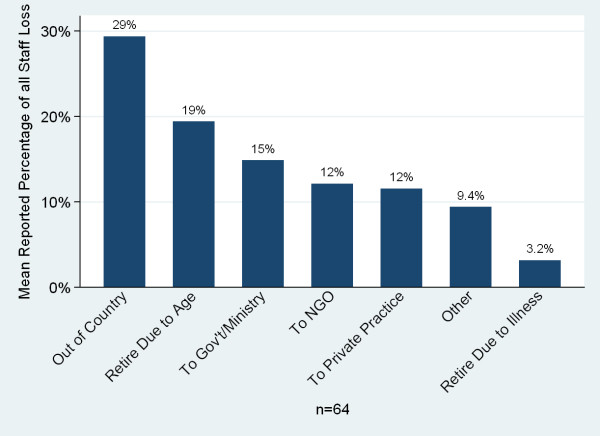
**Sub-Saharan African medical schools reasons for staff loss**.

Seventy-six percent of respondents (n = 71) reported a net gain of faculty over the past five years. Twenty-three percent reported a net loss (Figure 15). The most common reason given for staff loss was migration out of the country (mean 29.4%, n = 64) followed by retirement due to age, loss to government/ministry jobs, loss to non-governmental organizations, and loss to private practice (Figure 16).

Respondents reported a number of medical school measures to support faculty research including internal research and training programs (78%, n = 96), strengthened institution research tools (66%), funding for external research training (58%), research funding support (57%), and funded research time (29%).

**Figure 17:**
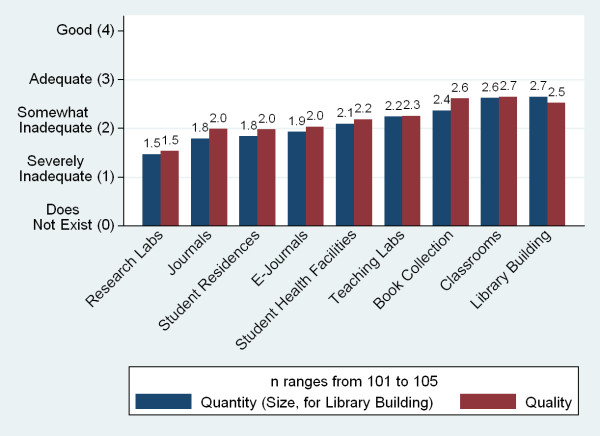
**Adequacy of student and teaching resources**.

**Figure 18:**
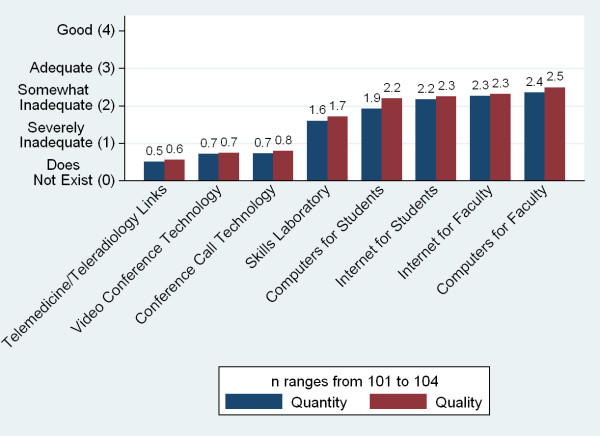
**Adequacy of technology resources**.

**Figure 19:**
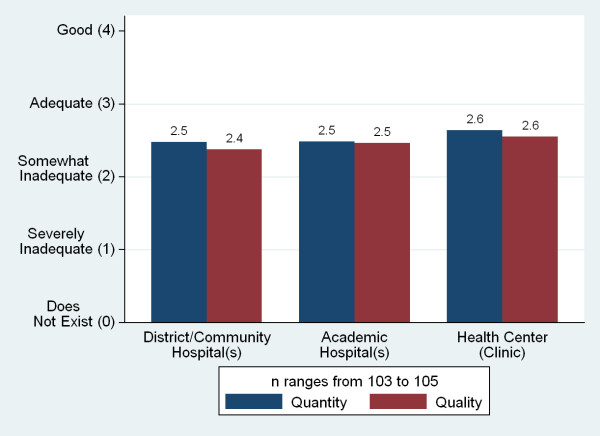
**Adequacy of clinical teaching sites**.

The quantity and quality of a number of resources--student and teaching resource, technology, and clinical teaching sites--were perceived by respondents on average to be below adequate (Figures 17, 18 and 19). With the exception of the size of the library, there was no single resource for which more than a quarter of schools described either the quality or quantity of the resource to be 'good'. The greatest insufficiencies were seen in research and skills labs, journals and e-journals, student residences, student computers and advanced technology resources (telemedicine and video conference and conference call technology). The most often cited reasons for not using the internet for teaching were lack of infrastructure and funds (90%, n = 49), lack of information technology (IT) connections (69%), and lack of trained persons to support instruments (57%).

**Figure 20:**
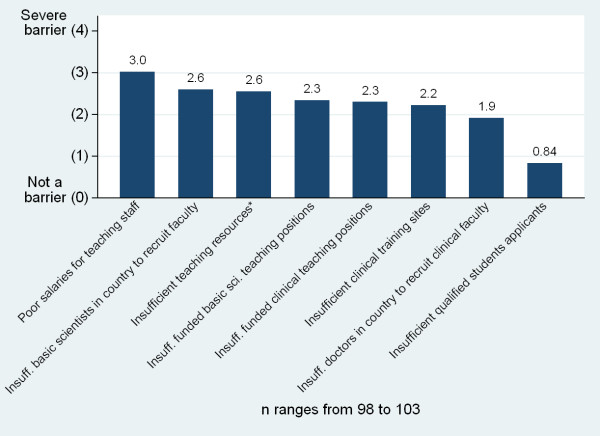
**Barriers to increasing the number of medical school graduates**.

**Figure 21:**
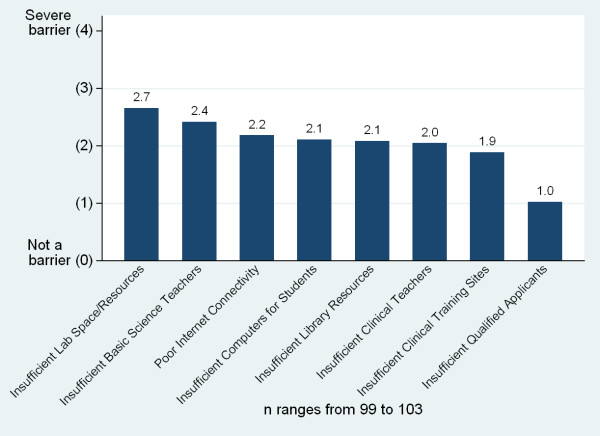
**Barriers to increasing the quality of medical school graduates**.

The perceived most severe barriers to increasing the number of graduates included poor salaries for teaching staff, insufficient number of basic scientists in the country from which to recruit faculty and insufficient teaching resources (Figure 20). The perceived most severe barriers to improving the quality of graduates included insufficient lab space and resources, insufficient teachers of basic sciences, poor internet connectivity and insufficient computers for students (Figure 21). In free response, the most frequently raised needs for increasing the quantity and quality of graduates were related to infrastructure and equipment issues, followed by faculty related issues and clinical training site issues (Table 2). However, faculty related issues were most commonly cited as the single greatest need in order to improve the quality of graduates while infrastructure related needs were most commonly cited as the greatest need to increase the quantity of graduates.

**Table 2:** Greatest needs for increasing the quantity and quality of medical school graduates

		Increase quality of graduates				Increase quantity of graduates			
		**Total** **answers**	**Greatest** **need**	**Second greatest need**	**Third greatest need**	**Total answers**	**Greatest need**	**Second greatest need**	**Third** **greatest need**

	**Total number of responses**	**281**	**94**	**94**	**93**	**277**	**94**	**94**	**89**

	**Total**	**91**	**28**	**38**	**25**	**96**	**37**	**38**	**21**
	
**Infrastructure and equipment issues**	General or multiple types	40	17	17	6	52	25	20	7
	
	Labs	14	7	4	3	10	3	5	2
	
	Computers/ICT	18	1	7	10	6	0	3	3
	
	Teaching aids/resources	8	2	4	2	12	4	7	1
	
	Libraries	4	1	2	1	4	0	1	3
	
	Other	7	0	4	3	12	5	2	5

	**Total**	**84**	**35**	**25**	**24**	**77**	**30**	**27**	**20**
	
**Faculty-related issues**	General or quantity	32	12	10	10	50	24	19	7
	
	Salary/Quality of life issues	23	6	6	11	12	1	3	8
	
	Basic science faculty	10	4	4	2	5	0	2	3
	
	Training/pedagogy	7	5	2	0	1	0	0	1
	
	Faculty quality	6	4	1	1	3	1	1	1
	
	Clinical faculty	3	2	1	0	2	2	0	0
	
	Other	3	2	1	0	4	2	2	0

**Clinical training sites**	**Total**	**25**	**3**	**15**	**7**	**33**	**9**	**15**	**9**
	
	General	12	2	6	4	14	3	8	3
	
	Academic hospital	13	1	9	3	16	6	7	3
	
	Clinics	0	0	0	0	3	0	0	3

**Budgetary issues**	**Total**	**21**	**10**	**2**	**9**	**22**	**6**	**4**	**12**
	
	General	14	9	0	5	17	5	3	9
	
	Student aid/grants	3	0	0	3	4	1	1	2
	
	Governmental support	4	1	2	1	1	0	0	1

**Curricular issues**	**Total**	**21**	**7**	**3**	**11**	**5**	**3**	**0**	**2**
	
	General/Aligned with needs	12	5	2	5	4	3	0	1
	
	Community-based/								
	Problem-based	5	1	1	3	0	0	0	0
	
	PGME	2	1	0	1	1	0	0	1
	
	Other	2	0	0	2	0	0	0	0

**Other**	**Total**	**39**	**11**	**11**	**17**	**44**	**9**	**10**	**25**
	
	Secondary education/								
	admissions policies	7	3	1	3	11	2	5	4
	
	Research	10	3	3	4	2	0	0	2
	
	Linkages/Cooperation	3	0	1	2	6	0	0	6
	
	Administrative reform Inside								
	school	5	2	2	1	3	0	1	2
	
	Understanding from gov't	0	0	0	0	8	5	0	3
	
	Student living conditions	3	0	0	3	4	1	1	2
	
	Other	11	3	4	4	10	1	3	6

Schools reported a number of initiatives to address barriers to increasing the quantity and quality of medical school graduates (Additional file 3). Common responses included construction of additional facilities, faculty recruitment and development, seeking donor support, developing institutional linkages, curriculum development, community-based education, use of technology for teaching, and strategies to reduce student failure (Table 3). Unique responses included: establishing a graduate entry medical education program, introducing a book bank available to all medical students, increasing internally generated revenue (such as through the operation of a Clinical Diagnostic Center or Fitness Center), and transferring supervision of the medical school from the Ministry of Education to the Ministry of Health.

**Table 3:** Focus of initiatives reported by respondents to address barriers to increasing the quantity and quality of medical school graduates

Issue addressed	Innovations named
Faculty	50

Infrastructure	34

Curriculum	27

Clinical sites	26

Student	23

Collaboration	14

Funding	12

Other	32

**Figure 22:**
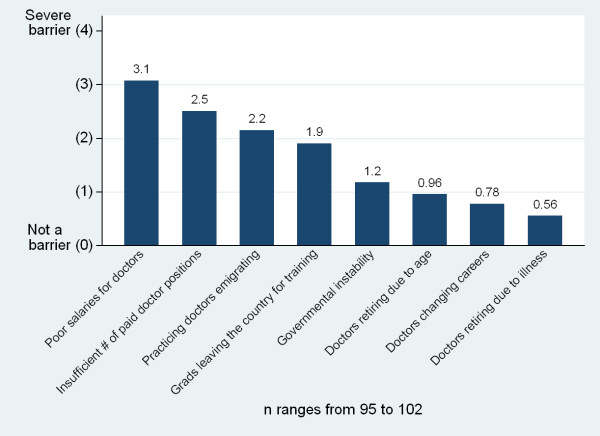
**Barriers to increasing the number of medical doctors in the country**.

The survey also examined perceived barriers to increasing the number of medical doctors in the country and medical school initiatives to improve medical doctor retention (Figure 22). The most commonly reported strategies to improve retention included raising salaries for faculty at the University, launching or strengthening post-graduate medical education programs, and launching or strengthening community-based education programs (Table 4). However, 27% of respondents (n = 100) indicated they had taken no steps to address doctor retention, an additional 9% explicitly stated retention was an issue which the government should address rather than the school, and 13% responded by listing strategies undertaken at the national level rather than the school level.

**Table 4:** Medical school strategies implemented to improve medical doctor retention in Country

Strategies implemented to improve doctor retention in the country	No. of schools
Raising salaries for faculty at the University	20

Launching or strengthening PGME programs	13

Launching or strengthening CBE programs	9

Recruiting graduates as faculty/providing a career path	6

Providing research support inside the medical school	4

Lobbying the government to make changes	3

Other	5

### Multivariable analysis

Respondents were asked to rate the adequacy of the quantity and quality of twenty individual resources on a 0-4 Likert scale (Figures 17, 18 and 19). Those responses were aggregated into six 'resource scores'--scores for buildings, libraries, labs, clinical sites, internet, and advanced information and communications technology (ICT)--(Table 5) and checked for correlation with a series of independent variables. Higher GDP was associated with higher scores for five of the six resources, older schools had better scores for four, and public schools rated their resources as worse in three indicators. Schools charging higher tuition fees reported more advanced ICT resources (Table 6).

**Table 5:** Resource score composition

Medical school resource indices	Components
Building score	Library building (size & quality), Classrooms (quantity & quality), StudentrResidences (quantity & quality)

Library score	Library building (size & quality), Book collection (quantity & quality), Journals (quantity & quality), E-Journals (quantity & quality)

Clinical sites score	Academic hospital(s) (quantity & quality), District/Community hospitals (quantity & quality), Health centers/clinics (quantity & quality)

Laboratory score	Teaching labs (quantity & quality), Research labs (quantity & quality), Skills laboratory (quantity & quality)

Internet score	Computers for students (quantity & quality), Internet for students (quantity & quality), Computers for faculty (quantity & quality), Internet for faculty (quantity & quality)

Advanced ICT score	Conference call technology (quantity & quality), Video conference technology (quantity & quality), Telemedicine/Teleradiology links (quantity & quality)

**Table 6:** Associations with resource scores

Outcome variable	Possible range of variable	Statistically significant predictors	Effect size	95% Confidence intervals	*P-Value*
Building score	0 to 24	French as a language of instruction	-4.9	-6.8 to-3.0	*P < 0.0001*
		GDP per capita (in US$, log scale)	1.6	0.54 to 2.8	*P = 0.0047*

Library score	0 to 32	Age of medical school (in years)	0.14	0.075 to 0.20	*P < 0.0001*
		English as a language of instruction	6.3	3.3 to 9.2	*P < 0.0001*
		Public ownership of medical School	-6.0	-9.1 to-2.9	*P = 0.00024*
		GDP per capita (in US$, log scale)	2.3	0.0014 to 4.6	*P = 0.0499*

Clinical sites score	0 to 24	Age of medical school (in years)	0.059	0.011 to 0.11	*P = 0.015*
		Arabic as a language of instruction	4.1	0.60 to 7.5	*P = 0.022*

Laboratory score	0 to 24	Age of medical school (in years)	0.092	0.037 to 0.15	*P = 0.0015*
		GDP per capita (in US$, log scale)	2.8	0.85 to 4.7	*P = 0.0062*
		Public ownership of medical school	-3.2	-6.0 to-0.33	*P = 0.029*

Internet score	0 to 32	GDP per capita (in US$, log scale)	4.1	2.3 to 5.8	*P < 0.0001*
		Public ownership of medical school	-3.6	-6.9 to-0.24	*P = 0.036*

Advanced ICT score	0 to 24	Age of medical school (in years)	0.094	0.051 to 0.14	*P < 0.0001*
		Annual tuition (in thousands of US$)	0.71	0.35 to 1.1	*P = 0.00015*
		GDP per capita (in US$, log scale)	2.6	0.92 to 4.2	*P = 0.0032*

Analyses performed on school processes included examinations of the numbers of graduates, percentages of faculty positions vacant, percentage of gross faculty loss over the past five years, numbers of PGME programs, and percentages of faculty involved in research. Public schools (*P *= 0.0011), schools with larger faculties (*P *= 0.049), and schools with more advanced ICT (*P *= 0.017) produce more graduates per year. Private schools (*P *= 0.0092) and schools in countries with higher GDP per capita (*P *= 0.0035) have a lower percentage of faculty positions vacant. Schools that reported increasing faculty salaries or providing faculty bonuses to address barriers to enlarging the number of doctors trained have lost a smaller percent of their faculty over the past five years (*P *= 0.018). Older medical schools (*P *= 0.00090), schools in southern Africa (survey respondents from southern Africa included eight from South Africa and one from Botswana) (*P *= 0.016), and schools with more faculty involved in research (*P *= 0.0041) have more PGME programs.

Schools that provided faculty with strengthened institutional research tools (defined as technical support, access to journals, ethics committees, research committees, etc.) (*P *= 0.00015) and funded research time (*P *= 0.045) have greater faculty involvement in research. A statistically significant relationship was not seen for research funding support (e.g. for equipment, research supplies), internal research training programs for faculty, or funding to attend external research training programs. Schools with English as a language of instruction have faculties more engaged in research (*P *= 0.024) and schools with Arabic as a language of instruction have faculties less likely to engage in research (*P *= 0.031).

Additional analyses included the use of various teaching methods, estimated locations of doctors five years after graduation, and total numbers of medical schools in each country. The SAMSS survey asked about the extent of a school's use of team-based learning (TBL), community-based learning (CBL), and problem-based learning (PBL). In the preclinical years, a higher degree of any of these instructional methods made a school more likely to use any of the others (*P *< 0.0001), while in the clinical years, the use of TBL was correlated with a higher use of CBL (*P *< 0.0001) and PBL (*P *= 0.00026), but the association between PBL and CBL was not statistically significant (*P *= 0.055).

It was seen that doctors were more likely to be in rural general practice five years after graduation if they were from schools reporting compulsory service requirements (*P *= 0.039), if they were from schools offering a moderate number of PGME programs (1-5) rather than no or many PGME programs (*P *= 0.016), and if they were from francophone schools (*P *= 0.016). Graduates were also more likely to be in general practice, urban or rural, if they were from francophone schools (*P *= 0.0070). In the analysis of total numbers of medical schools, countries with larger populations (*P *< 0.0001) and greater land mass (*P *< 0.0001) were likely to have more medical schools. There was no statistically significant correlation seen with GDP per capita, national language, or region of Africa. The results of these analyses are summarized in Table 7.

**Table 7:** Significant associations in correlative analysis

Outcome variable	Observed range of variable	Statistically significant predictors	Effect size	95% Confidence intervals	*P-Value*
# of Graduates	4 to 384	Public ownership of medical achool Total number of teaching staff Advanced ICT score	64 0.068 3.9	27 to 102 0.00042 to 0.14 0.72 to 7.2	*P = 0.0011* *P = 0.049* *P = 0.017*

% of Teaching Staff Positions Vacant	0% to 86%	GDP per capita (in USD, log scale) Public ownership of medical school	-9.8% 16%	-17% to -3.0% 4.0% to 28%	*P = 0.0035* *P = 0.0092*

% of Faculty Involved in Research (by quartile: 0-1%, 1.6-5%, 10%-15%, 20%-100%)	1-4	Strengthened institutional research tools Provision of funded research time English as a language of instruction Arabic as a language of instruction	0.90 0.51 0.58 -0.81	0.45 to 1.35 0.022 to 1.0 0.067 to 1.6 -1.6 to-0.011	*P = 0.00015* *P = 0.049* *P = 0.027* *P = 0.047*

% Gross Faculty Loss over Past Five Years	0% to 61%	Raising salaries/providing bonuses named as innovation	-7.4%	-13% to -1.3%	*P = 0.018*

# of PGME programs (by tercile: 0, 1-5, or 6+)	1-3	Age of medical school (in years) From southern Africa Having faculty in research	0.017 0.64 0.61	0.0085 to 0.025 0.064 to 1.2 0.16 to 1.0	*P = 0.00090* *P = 0.030* *P = 0.0082*

Use of TBL, PBL, CBL	Ordinal variables, range 1-4	Preclinical years: TBL-PBL Preclinical years: CBL-PBL Preclinical years: TBL-CBL Clinical years: TBL-CBL Clinical years: TBL-PBL	*Chi squared test does not yield effect size*. *All associations seen were positive*.		*P < 0.0001* *P < 0.0001* *P < 0.0001* *P < 0.0001* *P = 0.00026*

% of Graduates Preferring Practice as Rural General Practitioners 5 Years out	0% to 42.7%	Existence of a compulsory service programs Moderate number of PGME programs French as a language of instruction)	7.1% 12% 9.8%	0.12% to 14% 4.5% to 19% 1.8% to 18%	*P = 0.039* *P = 0.016* *P = 0.016*

% of Graduates Preferring Practice as Any General Practitioner 5 Years Out	0% to 100%	French as a language of instruction	22%	6.1% to 37%	*P = 0.0070*

Total Number of Medical Schools in a Country	0 to 29	Population of country (millions) Total land mass of country (millions of km^2^)	0.13 4.3	0.095 to 0.17 2.6 to 6.0	*P < 0.0001* *P < 0.0001*

## Discussion

The findings of the SAMSS survey significantly expand the baseline knowledge of medical education in Sub-Saharan Africa. The list of medical schools generated is itself the most complete list of medical schools in the region and identifies over 60 new schools previously not listed by the World Health Organization or the Foundation for Advancement of International Medical Education and Research (FAIMER). The very number of medical schools identified demonstrates the need for current data on medical education in SSA. The findings of the SAMSS survey establish baseline training capacity indicators, identify critical barriers for capacity expansion and quality improvement, and identify innovative strategies for addressing barriers. These findings provide a building block for future evidence based medical education research, investment, and policy making.

Medical schools in SSA are expanding, both in overall number and enrolments. In the past 20 years, at least 58 new medical schools have begun training doctors. However, medical school enrolments remain relatively small with 39% of respondents reporting first year enrolment at or below 100 students. These relatively small intakes suggest an area to build on existing capacity. In fact, 73% of established respondent schools report recent enrolment increases, and 45% of respondents report plans to increase over the next five years. More than half of the schools (58%) report having mandates to expand, most often from Ministries of Education and Health. This expansion is critical for strengthening the health care workforce in the region, but it also presents significant challenges for medical schools and national health systems.

Medical schools in SSA are also offering post-graduate training opportunities with over half of respondents reporting post-graduate training programs. Increasing the number of residency posts will increase in-country training opportunities, boosting the number of specialists in the country and the pool of potential junior faculty to recruit from.

Growth has also placed a strain on medical school infrastructures. Over half of the respondents reporting plans to expand indicate that they are unlikely to reach their goal enrolment numbers. Medical schools report inadequacies in a number of key physical resource areas, including skills and research labs, journals, student residences, and computers. Expansion further taxes these scarce resources. The most significant reported barriers to improving quality and increasing graduate numbers are insufficient physical infrastructure (labs, computers, teaching resources, and libraries) and faculty shortages. Faculty shortages include both basic science and clinical faculty, and many respondents attribute shortages to salary and quality of life issues. Of note, an insufficient number of qualified applicants is not seen as a barrier at the majority of medical schools.

Schools have implemented a number of strategies to address inadequacies and barriers to expansion. Strategies generally focus on addressing insufficient physical infrastructure, increasing faculty numbers through recruitment and faculty development strategies, maximizing existing resources through the use of technology, and developing external partnerships both locally and on an international level to provide clinical teaching sites, donor support, and educational and research exchanges. A number of schools also report unique strategies such as establishing a graduate entry medical program, increasing internally generated revenues through clinical services or the operation of a fitness centre, and transferring the supervision of the medical school from the Ministry of Education to the Ministry of Health. All of these strategies will need further evaluation but the compilation and sharing of these strategies provides an important opportunity for institutions to develop and adopt successful strategies, and collaborate to develop evidence based approaches.

Schools also report a number of strategies to improve medical doctor retention in their countries. The most common strategies include increasing salaries for faculty, establishing post-graduate medical education (PGME) programs, establishing community based education, recruiting graduates as faculty, establishing career pathways, and strengthening research support for faculty. While multivariable analysis showed few significant correlations between these strategies and reduced migration of graduates out of country, schools that provided bonuses or increased salaries for their faculty appeared to have less attrition of their own teaching staff. Additionally, faculty research, which is important for faculty retention, is significantly supported by funded research time for faculty members and strengthened institutional research tools (administrative and technical support, access to journals, and research and ethics committees).

Many schools report focused recruitment of rural students and student preparatory programs--two strategies often used to improve access to medical education for rural students and improve overall rural retention of medical doctors [18]. Sixty-nine percent of schools report compulsory service requirements for graduates in their country. While it stands to reason that compulsory service programs increase the availability of doctors in rural areas, this study provides the first continent-wide evidence that the existence of compulsory service programs increases the likelihood of future rural general practice. Schools with a moderate number of PGME programs (1-5), compared to those schools with no PGME programs and schools with many PGME programs (6-14) and French speaking schools, also demonstrate an increase likelihood of future rural general practice. These findings deserve further study to clarify the factors contributing to greater rural practice. What factors contribute to rural retention in Francophone countries and are these transferrable to other countries? Does the relationship of PGME to rural practice indicate the opportunity to pursue PGME in country decreases the likelihood of migration? Do a greater number of PGME programs relate to greater specialization and therefore decreased rural practice? These findings suggest important distinctions in strategies to address rural retention.

In the free-response section of the survey, schools were asked about their needs/requirements for increasing the quantity and the quality of their graduates. Referring to graduate quality, the highest number of respondents (35 of 94) named faculty-related challenges as the greatest need. Referring to graduate quantity, infrastructure issues were most commonly named as the greatest need (37 of 94). Needs related to the medical curriculum were primarily seen as needs related to graduate quality and challenges related to clinical training sites were primarily seen as needs to address graduate quantity. This information can help guide investment by linking the type of investment with the desired result: investment in infrastructure and clinical sites help schools train more doctors while investment in faculty and curricula help improve the quality of the doctors trained.

Despite strategies to address doctor retention in country and in rural areas, migration remains a significant issue in most countries. On average, 27% of respondents' domestic graduates were reported as likely to migrate out of their country within five years of graduation, most often to countries outside of Africa. A concerning finding of this study is the number of medical schools (40) reporting no specific school-level steps to address doctor retention and the additional nine schools explicitly stating retention is an issue which the government should address rather than the school. While doctor retention must be addressed across the spectrum of education, national, and international policies, this study clearly shows that many schools are implementing strategies to increase retention, and medical school level strategies, such as PGME, are showing promise in improving retention, particularly in rural areas.

Additional findings that deserve attention are the rise of the private (for-profit and not-for-profit) medical schools and tuition costs for medical school. The first private medical schools responding to the survey were founded in the 1990s and they represent an area of significant growth and potential for medical education in the region. However, private medical schools also present a challenge to a health care system that has historically relied on public institutions for training. Issues of quality assurance and accreditation, relationships to government organizations, and cost will need to be investigated as these private institutions continue to grow. This study suggests there are models for managing these issues. For example, four out of the five medical schools in Tanzania are under private ownership, yet all five schools report accreditation by the national level Tanzania Commission for Universities. This is in sharp contrast to other SSA countries that report inconsistent accrediting practices even among public schools. A better understanding of the Tanzanian system may help guide countries where private medical schools are just beginning to develop.

Likely related to the growth of private medical schools are the increasing levels of tuition costs seen in SSA medical schools. Although costs may be seen as modest by international standards, this may not feel so for poorer students studying medicine. Eight schools charge over US$ 5000 per year. Private medical schools are particularly dependent upon tuition fees for revenue. The increasing cost of medical school may have unintended consequences, providing access to education only for wealthy students and minimizing the likelihood that graduates will remain in country or serve in poor or peripheral areas. Trends in tuition costs and the effects of these costs should be closely monitored and accounted for in strategies to address medical education capacity expansion and doctor retention.

### Limitations

There were a number of limitations in the conduct of this study. A number of questions were of a subjective nature, individual surveys included some unanswered questions, and some inconsistencies were seen in country level questions from schools within the same countries. Questions such as the proportion of income from various sources, reasons for staff loss, and graduates' emigration and practice choices were often best estimates by respondents rather than data-based answers. In pilot testing, it was apparent that such data was generally not available, and pilot respondents reported that more specific questions were likely to pose a significant challenge to respondents and reduce the likelihood of response. We were advised that deans or their nominees would be able to provide reasonable estimates based on their first-hand experience. The study specifically found only 18% of 67 respondents have established school tracking of graduates and an additional 13% have conducted a one-time graduate assessment study. This finding suggests improved tracking systems are needed in order to document the impact of strategies to build capacity to improve doctor retention and practice patterns.

Another limitation was unanswered questions within individual returned surveys. Some questions were understandably left blank by some respondents, as in the case of questions regarding graduates for schools that had yet to graduate students. In other cases, when questions were left unanswered, attempts were made to contact respondents to complete the questionnaires. The number of responses to each question (n) is reported for all findings. Inconsistent answers were also seen in country level questions in some countries with multiple responding schools. This was seen in both the question on compulsory service requirements and the setting of competencies or expected skill sets by the government or professional councils. The respondents' reports of compulsory service requirements are also inconsistent with other published country level reports of compulsory service programs [17]. These inconsistencies may be a result of differing interpretations of the questions, differing requirements at school and country levels, or they may suggest communication between government bodies and medical schools needs to be evaluated and strengthened.

Finally, while a 72% overall response rate for surveyed schools is a strong response rate for a survey study of this kind, the findings reflect only a portion of the existing medical schools in Sub-Saharan Africa. Response rates were lower in some countries, including the Democratic Republic of Congo, Madagascar and Sudan. In most cases, countries with a high number of identified schools had lower response rates but a high absolute number of schools responding. Furthermore, an additional 23 schools were identified after the study period closed, reducing the response rate for all identified schools in SSA to 62%. While six of these schools had only begun training medical doctors after the initiation of SAMSS, 17 were established medical schools.

## Conclusions

International organizations and national governments are increasingly recognizing the critical role of a sufficient and well trained health care workforce to improve health in Sub-Saharan Africa and the critical need for more doctors in Africa as part of a balanced workforce. Baseline data and evidence based strategies are essential to implementing successful policies to build medical education capacity (for scaling up both the number and quality of graduates) and improve in country and rural retention of health care workers. The Sub-Saharan African Medical School Study significantly expands baseline knowledge on medical education infrastructure, capacity, barriers to expansion, and relationship to external organizations. The greatest needs for medical schools relate to physical infrastructure and faculty shortage issues and many schools are implementing innovative strategies to address barriers and improve doctor retention.

A better understanding of the current status and needs of medical schools in the region will help to guide school level, national, and international policies to strengthen medical education and produce more and better doctors to serve local needs. The data collected can be put to use to advance medical education in SSA and serves as a building block for future data collection, analysis, and evidence based practices. Finally, the identification and sharing of strategies to address barriers to capacity building and quality improvement promise to equip educators and policymakers with better tools to build stronger and more stable medical workforces in the future.

## Competing interests

The authors declare that they have no competing interests.

## Authors' contributions

CC participated in the design, implementation and analysis of the study, and drafted the manuscript. EB carried out the survey implementation and participated in the design and analysis of the study and in the drafting of the manuscript. TW performed the statistical analysis and helped to draft the manuscript. SF and FM conceived the study and participated in the design, implementation of the study, and editorial work on the manuscript. FO participated in the design and implementation of the study. RG contributed to the statistical analysis and participated in the editorial work on the manuscript. JK and DD contributed to the design of the survey. DA, AH, AK, and EO participated in the design of the study and survey collection. All authors read and approved the final manuscript.

## Supplementary Material

Additional file 1 **Sub-Saharan Africa Medical Schools Study Survey Instrument**. Click here for file

Additional file 2 **Medical schools in Sub-Saharan Africa**. A listing of all medical schools identified in Sub-Saharan Africa through SAMSS. Click here for file

Additional file 3 **Strategies to address barriers to increasing the number and quality of doctors trained: Free-response answers**. Click here for file
