# Supplementary material for: A survey of Sub-Saharan African medical schools
Source: Hum Resour Health. 2012 Feb 24;10:4. doi: 10.1186/1478-4491-10-4 (PMC3311571; doi:10.1186/1478-4491-10-4)
Supplement: Additional file 1 — Sub-Saharan Africa Medical Schools Study Survey Instrument. [file 1478-4491-10-4-S1.PDF]

The Sub-Saharan African Medical Schools Study (SAMSS)
Survey Study of All Sub-Saharan African Medical Schools

QUESTIONNAIRE

Instructions: Please answer each of the following questions. We recognize you may not have all the information to answer each of the questions precisely, for those questions please provide your best estimate. We ask that you type your answers into the space provided and return the survey electronically when possible. If you have additional comments for any of the questions, please add them at the end of the question.

Please return the survey to: samss.study@up.ac.za

If you prefer to write in answers and return the survey by post, please contact us to make arrangements for this. If you have any questions or concerns, please contact Dr. Eric Buch at Tel: +27-12-354-2481 or Email: eric.buch@up.ac.za.

SAMSS Survey Content Key: The questions have been coded around 5 major categories. The coding relates to the reasoning behind adding the question to the survey. In many cases, questions fall within more than one category, and in that case, the question was coded according to the most significant reason behind including the question.

(yellow)	Basic Demographic / Environment – these questions aim to establish basic demographic and environmental information in order to gain an understanding of the current status and framework within which medical schools function
(blue)	Capacity – these questions both seek to understand the current capacity of medical schools as well as the challenges and barriers they face
(orange)	Retention – these questions look at issues and strategies around faculty retention at medical schools, physician retention within countries and priming medical students for future retention such as through recruitment programs.
(green)	Innovation – these questions seek to establish the extent with which well recognized innovations (such as PBL, CBL) are being implemented as well as seeking new innovations by medical schools
(purple)	Relation to Health Systems – these questions look at how the medical school and the health system interact in a bidirectional manner to improve health outcomes

GENERAL INFORMATION

1.	Name of Medical School:	
	Former Name(s):	
	Street Address:	
	Town or City:	
	Province:	
	Postal Code:	
	Country:	
	Tel (1):	
	Tel (2):	
	Fax:	

2.	Name of Head of Medical School:	
	Title(s):	
	Tel (1):	
	Tel (2):	
	Fax:	
	E-mail:	

3.	Survey Respondent Name:	
	Title(s):	
	Position in School:	
	Tel (1):	
	Tel (2):	
	Fax:	
	E-mail:	

SCHOOL CHARACTERISTICS

The following questions relate to the general characteristics of your medical school. The purpose of these questions is to gain an understanding of the internal and external financial and health system environment in which Sub-Saharan African medical schools function.

4.	Which YEAR did instruction begin in your medical education program for training medical doctors?	

5.	What is the LANGUAGE of instruction? (Choose all that apply)	
	English	
	French	
	Portuguese	
	Arabic	
	Other (Please specify):	

6.	a. What is the OWNERSHIP of your medical school?	
	Public	
	Private Not-For-Profit, Faith-Based	
	Private Not-For-Profit, Non-Faith-Based	
	Private For Profit	
	Other (Please specify):	
	b. Is your medical school part of a University?	
	Yes	
	No	
	c. If yes, what is the name of the University?	

7.	What PROPORTION of your medical school's annual INCOME* comes from each of the following sources? (Approximates)<br><br>* Annual income includes monetary income from sources such as government, donors, tuition and fees, research and grants, clinical services, etc.	
	Ministry of Education:	%
	Ministry of Health:	%
	Provincial or Regional Government	%
	District or Local Government	%
	Local / National Donors:	%
	International Donors:	%
	Student Paid Tuition and Fees:	%
	Research and Grants:	%
	Clinical Services from Faculty Practice*:	%
	Other (Please specify):	%
		%
		%
	**TOTAL**	**100%**

* Faculty Practice - a university owned medical practice aimed at providing additional income to academic staff and the university, and also to provide an additional teaching platform for undergraduate / postgraduate students and other health professionals.

8.	What PROPORTION of your medical school's annual EXPENDITURES is for each of the following? (Approximates)	
	PROFESSIONAL PERSONNEL<br><br>Professional Personnel includes teaching staff, research professionals, executive administration manager	%
	NON-PROFESSIONAL PERSONNEL<br><br>Non-Professional Personnel includes administrative staff, technical employees, general service employees	%
	GOODS AND SERVICES<br><br>All expenditure on buying goods and services needed for the daily operations of the school. Examples of goods include toilet paper, stationery, small equipment etc.; services include cleaning, food, security services, all contracted services etc.	%
	CAPITAL EQUIPMENT<br><br>This includes fixed and movable (medium to large) medical, mechanical, electrical, electronic, communication etc equipment plus the maintenance of buildings and infrastructure. It may also include new buildings or renovations.	%
	Other (Please specify):	%
		%
	**TOTAL**	**100%**

9.	a. What is the annual average TUITION/FEES per student per year? (If no tuition/fees costs, please indicate "0")	
	For nationals:	US$
	For foreigners:	US$
	b. In addition to tuition and fees, what would you estimate to be the total annual EXPENSES for a student? (Including boarding, lodging, transportation, personal expenses)	US$

10.	a. Does your medical school have a written MISSION STATEMENT?<br><br>A Mission Statement is a brief statement of the aims, values, and overall plan of your medical school.	
	Yes	
	No	
	b. If yes, please write in your Mission Statement here or send us a copy by e-mail (send to: eric.buch@up.ac.za):	

11.	a. Does your medical school undergo a periodic accreditation or formal evaluation conducted by a body or organization external to your medical school?	
	Yes	
	No	
	b. If yes, what is the name of the body or organization?	

12.	How often does your medical school undergo a SELF-ASSESSMENT* process? (If your school does not have an established self-assessment process, please indicate "none")<br><br>* Self-assessment refers to an established process within your medical school or university to assess the curriculum, activities and programs in terms of content, quality and output.	
	One Time Self-Assessment<br>(Please indicate year of self-assessment: ___________)	
	Continuous Self-Assessment	
	Annually	
	Every 2 Years	
	Every 5 Years	
	Other (Please specify):	
	None	

13.	Which of the following health worker education programs exist in your university/institution? If none, please indicate "none."	
	Nursing	
	Pharmacy	
	Dentistry	
	Health Officers/Clinical Officers	
	Public Health	
	Health Care Management	
	Other (Please specify):	
	None	

UNDERGRADUATE STUDENTS

The following questions relate to the undergraduate medical students at your medical school. The purpose of these questions is to develop a baseline understanding of the current capacity and characteristics of African medical students, as well as plans to increase class sizes.

14.	a. Do you have RECRUITMENT* programs focused on the following populations? (Choose all that apply or "none" if there are no specific recruitment programs)<br><br>* Recruitment can include any activity aimed at encouraging applications from specific populations.	
	Rural Students	
	Women	
	Special Groups (Please specify):	
	Other (Please specify):	
	None	
	b. What PROPORTION of your students undergo a preparatory program* prior to entering the first year medical school class? If your medical school does not offer a preparatory program, please answer zero ("0")<br><br>* Preparatory programs include training programs prior to medical school entry to specifically prepare students for the medical school curriculum and improve performance during the medical school years.	%

15.	Does your school designate a set number of student positions each year for the following populations? (Choose all that apply or "none" if there are no designated positions)	
	Rural Students	
	Women	
	Special Groups (Please specify):	
	Other (Please specify):	
	None	

16.	Does your school have a formal agreement/contract to train students from other countries? (If there are no agreements, please indicate "none")	
	Please list the countries:	
	None	

17.	a. How many students APPLIED to enter your current first year medical school class? (Or the latest available data, specify year: ____________)		
		Men	Women
	Nationals:		
	Foreigners:		
	If the admission process occurs outside of your medical school or university, please check here:		
	b. How many students ENROLLED in your current first year medical school class? (Or the latest available data, specify year: _____________)		
		Men	Women
	Nationals:		
	Foreigners:		
	c. How many students GRADUATED as medical doctors in 2008? (Or the latest available data, specify year: ______________)		
		Men	Women
	Nationals:		
	Foreigners:		

18.	a. What is the average proportion of your first year class who COMPLETE* their studies and graduate? (Approximate)<br><br>* Completion does not have to occur "on-time." If extra years of study are required, these graduates are still considered within the COMPLETED proportion.	%
	b. For those students who do not graduate from your medical school, what PROPORTION do not graduate for the following reasons?	
	School policy to reduce the class size after the 1st or 2nd year	%
	Transfer to another school	%
	Student Failure	%
	Other (Please specify):	%
		%
	**TOTAL**	**100%**

19.	a. Compared to 5 years ago, what has been the PERCENT CHANGE in number of students in your medical school first year class? Please indicate a percent and mark increase or decrease. If no change, please answer zero ("0").	%	Increase<br><br>Decrease

20.	a. Does your medical school plan to increase the number of students in the first year class within the next 5 years?	
	Yes	
	No	
	b. If yes, what is the GOAL number for first year medical school students in 5 years time?	
	c. How likely do you think your school is to reach this goal number? Please circle your answer.	

	Unlikely to Increase Numbers	Will Likely Reach 25% of Goal Number	Will Likely Reach 50% of Goal Number	Will Likely Reach 75% of Goal Number	Very Likely to Reach Goal Number

	d. Which of the following bodies have issued mandates to your medical school to increase enrollment? (Choose all that apply, if there is no external mandate to increase numbers, please indicate "none")	
	Ministry of Health	
	Ministry of Education	
	Provincial or Regional Government	
	District of Local Government	
	Other (Please specify):	
	None	

21.	Which of the following are medical school graduates required to complete before entering practice in your country? (Choose all that apply)	
	Internship	
	Post-graduate training	
	Compulsory/Community service	
	Other (Please specify):	

TEACHING STAFF

The following questions relate to the faculty who teach medical students in your medical school. Faculty is a critical resource in medical schools. The purpose of these questions is to gain a baseline understanding of the current teaching workforce along with the barriers and challenges to strengthening this group.

22.	a. What is the total number of teaching staff at your medical school?<br><br>Teaching staff includes all individuals with teaching responsibilities for medical students, including full-time, part-time and volunteer staff.	
	b. What PERCENT of your TOTAL AVAILABLE teaching staff positions is currently UNFILLED?	%
	c. What is the PROPORTION of each of the following teaching staff? (Approximates)	
	Men:	%
	Women:	%
	Nationals:	%
	Foreigners:	%

23.	a. How is your teaching staff paid? Fill in the PERCENT of your teaching staff in each category. (Approximates)	
	Primarily paid by the Medical School:	%
	Primarily paid by the Teaching Hospital:	%
	Primarily paid through private practice:	%
	Primarily paid by an organization outside of the medical school/teaching hospital	%
	Other (Please specify):	%
		%
	**TOTAL**	100%
	b. What PERCENT of your teaching staff primarily paid by the medical school or teaching hospital supplements their income through private practice?	%

24.	a. Compared to 5 years ago, what has been the GAIN and LOSS of total teaching staff? Please fill in a number for both. (Approximates)	
	GAIN	
	LOSS	
	b. Of those who left over the past 5 years, what PROPORTION of your school's teaching staff do you estimate left for the following reasons?	
	Move to Ministry/Government position:	%
	Move to non-governmental organization:	%
	Move to private practice	%
	Move out of country:	%
	Retirement due to age:	%
	Retirement due to illness:	%
	Other (Please specify):	%
		%
	Unknown:	%
	**TOTAL:**	**100%**

25.	a. What PROPORTION of your current teaching staff is involved in grant-supported or other funded/commissioned research activities?	%
	b. What measures has your medical school implemented to support research activities by your teaching staff? (Choose all that apply)	
	Funded Research Time	
	Research Funding Support e.g. for equipment, research supplies	
	Internal Research Training Programs for Faculty	
	Funding to Attend External Research Training Programs	
	Strengthened Institutional Research Tools*	
	Other (Please specify):	

* Research Tools include administrative and technical support, access to journals, Ethics Committees, Research Committees, etc.

CURRICULUM

The following questions relate to curriculum within your medical school. The purpose of these questions is to gain a better understanding of the curricular innovations African medical schools are implementing.

26.	a. How many years of study are required to graduate from your medical school?	

27.	To what extent is community-based learning utilized in the curriculum? Choose all that apply.<br><br>Community-based learning = a form of instruction where trainees learn professional competencies in a community setting focusing on population groups and also individuals and their everyday problems					
	Not At All	Occasionally	Frequently in Preclinical Courses	Frequently in Clinical Rotations	Extensively in Preclinical Courses	Extensively in Clinical Rotations

28.	To what extent is multi-disciplinary team-based learning utilized in the curriculum? Choose all that apply.<br><br>Multi-disciplinary team-based learning = an instructional approach aimed at preparing students to effectively work within a health care team that includes other health professionals, such as nurses, clinical officers, pharmacists, and others.					
	Not At All	Occasionally	Frequently in Preclinical Courses	Frequently in Clinical Rotations	Extensively in Preclinical Courses	Extensively in Clinical Rotations

29.	To what extent is problem-based learning (PBL) utilized in the curriculum? Choose all that apply.<br><br>Problem-based learning (PBL) = an instructional approach which uses priority health problems that medical practitioners are likely to encounter in their professional lives, as stimuli for learning. This approach emphasizes active self-directed learning by students individually or in small groups.					
	Not At All	Occasionally	Frequently in Preclinical Courses	Frequently in Clinical Rotations	Extensively in Preclinical Courses	Extensively in Clinical Rotations

30.	a. Are students required to undertake research projects in order to graduate?	
	In Preclinical	
	In Clinical	
	Not Required	
	b. Is a research report or thesis required in order to graduate?	
	Yes	
	No	

RESOURCES AND FACILITIES

The following questions are related to the state of resources at your medical school. Resources, including teaching resources, facilities, information technology and clinical teaching sites, are a critical component of teaching medical students. The purpose of these questions is to develop a baseline understanding of the current state of these resources.

31.	Please rate the adequacy of the following student/teaching resources.					
		Does not exist	Severely Inadequate	Somewhat Inadequate	Adequate	Good
	Library Building:					
	Size	1	2	3	4	5
	Quality	1	2	3	3	5
	Book Collection:					
	Quantity	1	2	3	4	5
	Quality	1	2	3	4	5
	Journals:					
	Quantity	1	2	3	4	5
	Quality	1	2	3	4	5
	E-Journals:					
	Quantity	1	2	3	4	5
	Quality	1	2	3	4	5
	Classrooms:					
	Quantity	1	2	3	4	5
	Quality	1	2	3	4	5
	Teaching Labs:					
	Quantity	1	2	3	4	5
	Quality	1	2	3	4	5
	Research Labs:					
	Quantity	1	2	3	4	5
	Quality	1	2	3	4	5
	Student Residences:					
	Quantity	1	2	3	4	5
	Quality	1	2	3	4	5
	Student Health and Wellness Facilities:					
	Quantity	1	2	3	4	5
	Quality	1	2	3	4	5

32.	Please rate the adequacy of the following technology resources.					
		Does not exist	Severely Inadequate	Somewhat Inadequate	Adequate	Good
	Skills Laboratory:					
	Quantity	1	2	3	4	5
	Quality	1	2	3	4	5
	Computers for Students:					
	Quantity	1	2	3	4	5
	Quality	1	2	3	4	5
	Internet for Students:					
	Quantity	1	2	3	4	5
	Quality	1	2	3	4	5
	Computers for Faculty:					
	Quantity	1	2	3	4	5
	Quality	1	2	3	4	5
	Internet for Faculty:					
	Quantity	1	2	3	4	5
	Quality	1	2	3	4	5
	Conference Call Technology:					
	Quantity	1	2	3	4	5
	Quality	1	2	3	4	5
	Video Conference Technology:					
	Quantity	1	2	3	4	5
	Quality	1	2	3	4	5
	Telemedicine/Teleradiology Links:					
	Quantity	1	2	3	4	5
	Quality	1	2	3	4	5

33.	Please rate the adequacy of the following clinical teaching and research sites/platforms for your medical school.					
		Does not exist	Severely Inadequate	Somewhat Inadequate	Adequate	Good
	Academic Hospital(s):					
	Quantity	1	2	3	4	5
	Quality	1	2	3	4	5
	District / Community Hospital(s):					
	Quantity	1	2	3	4	5
	Quality	1	2	3	4	5
	Health Center (Clinic):					
	Quantity	1	2	3	4	5
	Quality	1	2	3	4	5

34.	a. Does your medical school use the internet to augment your teaching?	
	Yes	
	No	
	b. If yes, what type of internet learning does your school use (Choose all that apply)?	
	Video Distance Lecturing	
	E-curriculum (Online curriculum)	
	Other (Please specify):	
	c. If no, what is the reason for not using internet learning (Choose all that apply)?	
	Lack of infrastructure and funds	
	Lack of trained persons to support instruments	
	Lack of IT connections	
	Other (Please specify):	

POST-GRADUATE (RESIDENCY) TRAINING

The following questions relate to post-graduate training and retention in your country. Post-graduate training is a critical contributor to the medical doctor workforce in a country. The purpose of these questions is to gain an understanding of the current availability of post-graduate training and the state of medical doctor retention in your country.

35.	a. Are the following post-graduate (residency) training programs offered at your institution?<br>b. If yes, how many graduates of each program are there each year?<br>c. Please indicate if the post-graduate (residency) training program is offered at other institutions in your country.					
		a. Offered at YOUR institution?		b. Total Number of Residents Graduating per year from YOUR institution	c. Offered at OTHER institutions in country?	
	Internal Medicine	Y	N		Y	N
	Medical Sub-Specialties*	Y	N		Y	N
	General Surgery	Y	N		Y	N
	Surgical Sub-Specialties**	Y	N		Y	N
	Obstetrics and Gynecology	Y	N		Y	N
	Pediatrics	Y	N		Y	N
	Psychiatry	Y	N		Y	N
	General / Family Practice	Y	N		Y	N
	Anesthesia	Y	N		Y	N
	Radiology	Y	N		Y	N
	Pathology	Y	N		Y	N
	Other (Please specify):	Y	N		Y	N
		Y	N		Y	N
		Y	N		Y	N
		Y	N		Y	N

* Medical Sub-Specialties include: Cardiology, Clinical Hematology, Dermatology, Endocrinology, Gastroenterology, Geriatric Medicine, Medical Oncology, Nephrology, Neurology, Occupational and Physical Medicine, Pulmonology, Critical Care

** Surgical Sub-Specialties include: Cardiothoracic Surgery, Maxillo-Facial and Oral Surgery, Neurosurgery, Ophthamology, Orthopedic Surgery, Otorhinolaryngology, Pediatric Surgery, Plastic and Reconstructive Surgery, Urology, Vascular Surgery

36.	a. 5 years after graduation, what proportion of your domestic* graduates have MIGRATED out of your country?<br><br>* Domestic indicates these graduates were originally from your own country.	
	To other African countries	%
	Outside of Africa	%
	b. Of those that remain after 5 years of graduation, what is the general preference for professional practice? (Please estimate to the best of your ability)	
	General Practice – Private, Urban	%
	General Practice - Private, Rural	%
	General Practice – Public Sector, Urban	%
	General Practice – Public Sector Rural	%
	Specialist (in or completed training) - Urban	%
	Specialist (in or completed training) - Rural	%
	Doctors who have left the profession	%
	Other (Please specify):	%
	**TOTAL**	100%
	c. For the questions above, what are these numbers based upon?	
	Established School Tracking of Graduates	
	One Time Graduate Assessment Study	
	Estimates	

37.	a. Are national licensing examinations beyond University examinations required for medical doctors to practice in your country?	
	Yes	
	No	
	b. Are additional examinations beyond your University required to practice as a specialist in your country?	
	Yes	
	No	

38.	a. Is COMPULSORY/COMMUNITY SERVICE required by your government following graduation for medical students?	
	Yes	
	No	
	b. If yes, how many years of COMPULSORY/COMMUNITY SERVICE are required?	Years
	c. What are the conditions of service for Compulsory/Community Service (Choose all that apply)	
	Paid Public Sector Work – Urban	
	Paid Public Sector Work – Rural	
	Unpaid Public Sector Work – Urban	
	Unpaid Public Sector Work – Rural	
	Other (Please specify):	
	d. Are select groups exempt from compulsory/community service?	
	Yes	
	No	
	e. If yes, which groups are exempt? Please fill in:	

External Organizations

The following questions relate to organizations external to your medical school. The purpose of these questions is to identify organizations that play an important role in setting priorities and collaborating with African medical schools.

39.	To what extent does each of the following groups participate in setting your medical school's priorities?					
		Not At All	Contributes to a minor degree	Contributes	Contributes Significantly	Primary Driver of Priorities
	Ministry of Health	1	2	3	4	5
	Ministry of Education	1	2	3	4	5
	Community Organizations	1	2	3	4	5
	Professional Councils/Organizations	1	2	3	4	5
	Medical School Graduates/Alumni	1	2	3	4	5
	International Affiliates/Partners	1	2	3	4	5
	Other (Please specify):					
		1	2	3	4	5
		1	2	3	4	5

40.	a. Do your country's government or professional councils set competencies required for all medical doctors in your country?	
	Yes	
	No – But they have a list of expected tasks and skills	
	No list of competencies or expected tasks/skills	
	b. If competencies or expected tasks/skills exist, to what degree does your school measure these competencies or tasks/skills? (choose one)	
	Measurement tools for ALL of the competencies/tasks/skills	
	Measurement tools for SOME of the competencies/tasks/skills	
	No measurement tools but faculty have a general idea of student competencies/skills	
	No measurement of government/professional council set competencies/skills	
	Other (Please specify):	

41.	How does your medical school participate in setting your country's health strategies and policies? If your medical school does not participate, please indicate "Does Not Participate".	
	School Officials sit on an official council or committee which advises the government ministries	
	School Officials submit written recommendations to the government ministries	
	School Officials informally advise the government ministries	
	Faculty research informs government policies	
	Other (please specify):	
	Does Not Participate	

42.	a. What kind of collaborations does your medical school have with other schools or organizations? Please indicate all types or if none, please indicate "none".	
	Student Exchange	
	Faculty Exchange	
	Research Funding/Collaborations	
	Intellectual/Academic Debates/Discussions/Colloquia	
	Other (please specify):	
	None	
	b. Please list your 5 MAIN collaborators both in your country and outside of your country.	
	IN COUNTRY:	
	Name	Nature of Collaboration
	1.	
	2.	
	3.	
	4.	
	5.	
	OUTSIDE THE COUNTRY:	
	Name	Nature of Collaboration
	1.	
	2.	
	3.	
	4.	
	5.	

43.	How many medical schools, which graduate medical doctors, are there in total in your country?	
	Public	
	Private	

Barriers to Increasing the Number of Medical Doctors

The following questions relate to barriers to increasing the number and quality of medical school graduates and medical doctors in your country. The purpose of these questions is to identify significant barriers and key areas of need for increasing the number of medical doctors in your country.

44.	How important are each of these barriers to IMPROVING THE QUALITY of graduates from your medical school?					
		Not A Barrier				Severe Barrier
	Insufficient laboratory space and resources	1	2	3	4	5
	Insufficient library resources	1	2	3	4	5
	Insufficient computers for students	1	2	3	4	5
	Poor internet connectivity	1	2	3	4	5
	Insufficient clinical training sites – hospitals, health centers	1	2	3	4	5
	Insufficient basic science teachers	1	2	3	4	5
	Insufficient clinical teachers	1	2	3	4	5
	Insufficient qualified students applying for medical school (insufficient strength of secondary education)	1	2	3	4	5
	Other (Please specify):	1	2	3	4	5
		1	2	3	4	5
		1	2	3	4	5

45.	How important are each of these barriers to INCREASING THE NUMBER of graduates from your medical school?					
		Not A Barrier				Severe Barrier
	Insufficient teaching resources – classrooms, laboratory space, library, computers	1	2	3	4	5
	Insufficient clinical training sites – hospitals, health centers	1	2	3	4	5
	Insufficient number of funded basic science teaching positions	1	2	3	4	5
	Insufficient number of funded clinical teaching positions	1	2	3	4	5
	Insufficient basic scientists in country to recruit basic science teaching staff	1	2	3	4	5
	Insufficient medical doctors in country to recruit clinical teaching staff	1	2	3	4	5
	Poor salaries for teaching staff	1	2	3	4	5
	Insufficient qualified students applying for medical school	1	2	3	4	5
	Other (Please specify):	1	2	3	4	5
		1	2	3	4	5
		1	2	3	4	5

46.	How important are each of these barriers to INCREASING THE NUMBER of medical doctors IN YOUR COUNTRY?					
		Not A Barrier				Severe Barrier
	Poor salaries for medical doctor positions	1	2	3	4	5
	Insufficient number of paid medical doctor positions	1	2	3	4	5
	Graduates leaving the country for post-graduate training	1	2	3	4	5
	Practicing doctors migrating out of the country	1	2	3	4	5
	Medical doctors changing careers/leaving medicine	1	2	3	4	5
	Medical doctor retirement due to age	1	2	3	4	5
	Medical doctor retirement due to illness	1	2	3	4	5
	Government instability	1	2	3	4	5
	Other (Please specify):	1	2	3	4	5
		1	2	3	4	5
		1	2	3	4	5

47.	In order of importance, what are the 3 greatest NEEDS/REQUIREMENTS for IMPROVING THE QUALITY of graduates from your medical school. Please explain each further. 1 = MOST IMPORTANT
	1.
	2.
	3.

48.	In order of importance, what are the 3 greatest NEEDS/REQUIREMENTS for INCREASING THE NUMBER of graduates from your medical school. Please explain each further. 1 = MOST IMPORTANT
	1.
	2.
	3.

49.	Please list INNOVATIONS developed and implemented at your medical school to address the barriers and challenges to increasing the number of medical doctors trained in your country
	1.
	2.
	3.

50.	*The following questions relate to medical doctor retention within your country.*<br>a. Is there a problem with medical doctor retention in your country? Please explain.
	b. What strategies has your medical school implemented to improve medical doctor retention in your country?

Thank you very much for answering this questionnaire. Should you have any questions, please contact us AND please remember to send additional documents (e.g. mission statement, written mandates, annual reports) to the address below.

Please return the survey to: samss.study@up.ac.za

Please also include you Bank Name, Branch or Branch Code, Account Type and Account Number to enable us to deposit your Honorarium at the earliest opportunity after receipt of the completed questionnaire.

Dr. Eric Buch
School of Health Systems and Public Health
University of Pretoria
PO Box 667 Pretoria, 0001, South Africa
Tel: +27-12-354-2481
Fax: +27-12-354-1750
Email: eric.buch@up.ac.za
