# Supplementary material for: A survey of Sub-Saharan African medical schools
Source: Hum Resour Health. 2012 Feb 24;10:4. doi: 10.1186/1478-4491-10-4 (PMC3311571; doi:10.1186/1478-4491-10-4)
Supplement: Additional file 2 — Medical schools in Sub-Saharan Africa. A listing of all medical schools identified in Sub-Saharan Africa through SAMSS. [file 1478-4491-10-4-S2.PDF]

Medical Schools in Sub-Saharan Africa

Note: A school is considered a medical school if it was actively training undergraduate medical doctors as of the summer of 2010. Ownership and Founding Date (the date a school first started training medical doctors) are listed for all medical schools that responded to the SAMSS survey. The schools that the SAMSS team attempted to survey but did not receive a response from (either because the school did not successfully receive the survey or because it did not complete a received survey) are marked with one asterisk (*). Schools that began to admit medical students or were identified after the close of the survey period (December, 2009) are marked with two asterisks (**).

Country	School Name	Ownership	Founding Date
Angola	Faculdade de Medicina, Agostinho Neto	Public	1963
	Universidade Jean Piaget de Angola	*	*
	Universidade Jose Eduardo dos Santos	**	**
	Universidade Mandume Ya Ndemofayo	**	**
	Universidade Lueji	**	**
	Faculdade de Medicina Universidade Katyavala Bwila	**	**
	Universidade Onze de Novembro	**	**
Benin	Faculté de Médecine, Université de Parakou	Public	2001
	Faculté des Sciences de la Santé de Cotonou	Public	1968
Botswana	University of Botswana School of Medicine	Public	2009
Burkina Faso	Institut Supérieur des Sciences de la Santé	Public	2005
	Ecole Supérieure des Sciences de la Santé, Université d'Ouagadougou	*	*
Burundi	Faculty of Medicine, University of Burundi	Public	1968
Cameroon	Faculté de Médecine et des Sciences Biomédicales, Université de Yaoundé	Public	1969
	Faculté de Médecine et des Sciences Pharmaceutiques de Douala	Public	2006
	Université des Montagnes	Private Not for Profit	2000
	Université de Buea	*	*
Central African Republic	Faculté de Sciences de la Santé de l'Université de Bangui	Public	1976
Chad	University of N'djamena	*	*
Comoros	Ecole de Médecine et de Santé Publique	*	*
Republic of Congo	Université Marien Ngouabi de Brazzaville	Public	1975
Côte d'Ivoire	UFR Sciences Médicales d'Abidjan	Public	1962
	UFR Sciences Médicales de Bouake	Public	1997
Democratic Republic of Congo	Faculté de Médecine, Université Catholique de Bukavu	Private Not for Profit	1990
	Goma University	Public	1994
	Université Adventiste de Lukanga	**	**
	Université Catholique de Bandundu	**	**
	Université Catholique de Graben	**	**
	Université Chrétienne Internationale	**	**
	Université Chrétienne Kinshasa	**	**
	Université Evangélique en Afrique de Bukavu	**	**
	Université de Kinshasa	*	*
	Université de Kisangani	*	*
	Université Kongo	*	*
	Université Mbujimayi	*	*
	Université Notre Dame du Kasayi	**	**

	Université Protestante au Congo	**	**
	Université Protestante de Kimpese	**	**
	Université Simon Kimbangu	**	**
	Université Simon Kimbangu de Bukavu	**	**
	Université Technologique Bel Campus	**	**
	Université de Lubumbashi	*	*
Djibouti	Ecole de Médecine de Djibouti	**	**
Equatorial Guinea	Universidad Nacional de Guinea Ecuatorial	**	**
Eritrea	Orotta School of Medicine	Public	2004
Ethiopia	Adama University	*	*
	Arbaminch School of Medicine	Public	2009
	Defense Health College, Medical School	Public	No Response
	Faculty of Medicine, Addis Ababa University	Public	1963
	Faculty of Medicine, Bahir Dar University	Public	2007
	Haramaya University Medical Faculty	Public	2007
	Hawassa University College of Health Sciences	Public	2003
	Hayat Medical School	*	*
	Mekelle University College of Medicine and Health Sciences	Public	No Response
	School of Medicine, Gondar College of Medicine and Health Sciences	Public	No Response
	School of Medicine, Jimma University	Public	1983
	St. Paul's Millennium Medical School	Public	2008
Gabon	Faculté de Médecine et des Sciences de la Santé	Public	1972
Gambia	University of the Gambia Medical School	*	*
Ghana	School of Medical Sciences, Kwame Nkrumah Univ of Science and Tech	Public	1975
	School of Medical Sciences, University of Cape Coast	*	*
	University for Development Studies	**	**
	University of Ghana Medical School	Public	1964
Guinea	Faculté de Médecine Pharmacie et Odontostomatologie, Université de Conakry	Public	1967
	Faculté des Sciences Médicales, Université Kofi Annan de Guinée	Private for Profit	2006
	Université la Source	Private for Profit	2007
Guinea-Bissau	Raul Diaz Arguelles	Public	2005
Kenya	Moi University School of Medicine	Public	1990
	University of Nairobi	*	*
	Kenyatta University	**	**
Liberia	A.M. Dogliotti College of Medicine	Public	1968
Madagascar	Université d'Antananarivo	*	*
	Université de Mahajanga	*	*
Malawi	College of Medicine, University of Malawi	Public	1991
Mali	Faculté des Sciences de la Santé, Université Kankou Moussa	Private for Profit	2009
	Faculty of Medicine, Pharmacy and Odontostomatologie	Public	1969
Mauritania	Université de Nouakchott	**	**
Mauritius	Department of Medicine, Faculty of Science, University of Mauritius	Public	1997
	Sir Seewoosagur Ramgoolam Medical College	*	*
Mozambique	Faculty of Medicine, Eduardo Mondlane University	Public	1963
	Universidade Católica de Moçambique	Private Not for Profit	2000
	Universidade Lúrio	*	*
	Universidade Zambeze	*	*
Namibia	University of Namibia	**	**
Niger	Faculté des Sciences de la Santé, Université Abdou Moumouni	Public	1974
	Abia State University	*	*

Nigeria	Ahmadu Bello University	*	*
	College of Health Science, Ebonyi State University	Public	1992
	College of Health Sciences, Benue State University	Public	2005
	College of Health Sciences, Delta State University	Public	2001
	College of Health Sciences, Igbinedion University	Private for Profit	1999
	College of Health Sciences, Ladoke Akintola University of Technology	Public	1991
	College of Health Sciences, Nnamdi Azikiwe University, Nnewi Campus	Public	1987
	College of Health Sciences, Obafemi Awolowo University	Public	1972
	College of Health Sciences, Osun State University	Public	2007
	College of Health Sciences, University of Abuja	Public	2004
	College of Health Sciences, University of Ilorin	Public	1977
	College of Health Sciences, University of Port Harcourt	Public	1979
	College of Medical Sciences, University of Maiduguri	Public	1978
	College of Medicine, Ambrose Alli University Ekpoma	Public	1991
	College of Medicine, University of Ibadan	Public	1948
	College of Medicine, University of Nigeria, Enugu Campus	Public	1967
	Danfodiyo University	*	*
	Faculty of Medicine, Bayero University Kano	Public	1986
	Lagos State University College of Medicine	Public	1999
	Madonna University College of Medicine	Private Not for Profit	1999
	University of Benin	*	*
	University of Calabar	*	*
	University of Jos	*	*
	University of Lagos	*	*
Rwanda	Faculty of Medicine, National University of Rwanda	Public	1963
Senegal	Brighton International University School of Medicine	Private for Profit	2006
	Faculté de Médecine, Pharmacie et d'Odontologie, Université Cheikh Anta Diop	Public	1918
	Institut Privé de Formation et de Recherches Médicales de Dakar	Private for Profit	2009
	Saint Christopher Iba Mar Diop	Private Not for Profit	2000
Seychelles	University of Seychelles, American Institute of Medicine	-	-
Sierra Leone	College of Medicine and Allied Health, University of Sierra Leone	Public	1988
Somalia	Amoud Medical School	Private Not for Profit	2000
	Benadir University	Private Not for Profit	2002
South Africa	Faculty of Health Sciences, Stellenbosch University	Public	1956
	Faculty of Health Sciences, University of Cape Town	Public	1919
	Nelson R. Mandela School of Medicine	Public	No Response
	School of Medicine, Faculty of Health Sciences, University of the Free State	Public	1971
	University of Limpopo, Medunsa Campus	Public	1978
	University of Pretoria	Public	1943
	Walter Sisulu University	Public	1985
	Wits Medical School, University of the Witwatersrand	Public	1919
Sudan	Ahfad Medical School for Women	Private Not for Profit	1990
	Al Neelain University	Public	2009
	El-Razi Medical and Health College	*	*
	Faculty of Medicine, University of West Kordofan	*	*
	Faculty of Medicine and Health Sciences, Sinnar University	Public	1997
	Faculty of Medicine and Health Sciences, University of Elimam Elmahadi	Public	2008
	Faculty of Medicine and Health Sciences, University of Kassala	Public	1991
	Faculty of Medicine, Gadarif	Public	1998
	Faculty of Medicine, International University of Africa	Public	1998

	Faculty of Medicine, The National Ribat University	Private Not for Profit	2000
	Faculty of Medicine, Upper Nile University	*	*
	Faculty of Medicine, University of Bahr Gazal	*	*
	Faculty of Medicine, University of Bakt Ruda	*	*
	Faculty of Medicine, University of Dongola	Public	1997
	Faculty of Medicine, University of Elfashir	*	*
	Faculty of Medicine, University of Gezira	Public	1978
	Faculty of Medicine, University of Juba	*	*
	Faculty of Medicine, University of Khartoum	*	*
	Faculty of Medicine, University of Kordofan	Public	1991
	Faculty of Medicine, University of Medical Sciences and Technology	Private Not for Profit	1996
	Faculty of Medicine, University of Omdurman Islamic	*	*
	Faculty of Medicine, University of Red Sea	Public	1998
	Faculty of Medicine, University of Shendi	Public	1990
	Faculty of Medicine, University of Science and Technology	*	*
	National College for Medical and Technical Studies	*	*
	National College for Medical and Technical Studies	Private Not for Profit	2005
	Nile Valley University - Faculty of Medicine & Health Science	Public	2006
	Sudan International University	*	*
	University Alzaeim Al Azhari	Public	No Response
Tanzania	Hubert Kairuki Memorial University	Private Not for Profit	1997
	International Medical and Technological University	Private Not for Profit	1997
	Kilmanjaro Christian Medical College	Private not for Profit	1998
	School of Medicine, Muhimbili University of Health and Allied Sciences	Public	1968
	Weill Bugando University College of Health Sciences	Private Not for Profit	2003
Togo	Faculté Mixte de Médecine et de Pharmacie (FMMP), Université de Lome	Public	1970
Uganda	Gulu University Faculty of Medicine	Public	2004
	Mbarara University Medical School	Public	1989
	School of Medicine, Kampala International University	Private for Profit	2006
	School of Medicine, Makerere University College of Health Sciences	Public	1923
Zambia	School of Medicine, University of Zambia	Public	1966
Zimbabwe	College of Health Sciences, University of Zimbabwe	Public	1963
	National University of Science and Technology, Faculty of Medicine	*	*
