# Supplementary material for: A survey of Sub-Saharan African medical schools
Source: Hum Resour Health. 2012 Feb 24;10:4. doi: 10.1186/1478-4491-10-4 (PMC3311571; doi:10.1186/1478-4491-10-4)
Supplement: Additional file 3 — Strategies to address barriers to increasing the number and quality of doctors trained: Free-response answers. [file 1478-4491-10-4-S3.PDF]

Innovations Implemented to Address Barriers to Increasing Number and Quality of Doctors Trained: Eastern Africa
A group of specialists provided land for residential house construction, which made a big difference in staff retention.
Accelerated Training of Medical Doctors
Accelerated Training of Public Health Officers
Adoption of community oriented programmes
Arrangement with nearby hospitals for clinical teaching sites
Assume management of an existing hospital from the region so as to make it a university hospital
Collaboration with District Hospitals
Community based services and training at primary heath centers
Construction of a multi-purpose library for teaching purposes.
Construction of classrooms
Construction of classrooms
Construction of laboratories
Construction of student hostels
Continual review of medical curriculum
Continuous appraisal and review of curriculum
Coordination of assessment
Coordination of blocs and tutors
Created a structured questionnaire to evaluate the deficiencies and barriers
Creation of internet facilities for students
Creation of new medical schools
Curricular initiatives including PBL
Curricular initiatives, including a community based education program(CBTP), Team Training program (TTP) and Student research program (SRP)
Develop necessary expertise in curriculum development to update curriculum with special emphasis on community-based medical training and evidence-based clinical studies.
Development and implementation of community medical oriented programs such as PBL,COBES
Development of an Educational development centre
Development of clinical training sites – hospitals, health centers and clinics available in the city to train students and graduates
Development of protocols and collaboration inside and outside the country to provide financial and technical support
Development of trainers with necessary teaching skills, capable of delivering quality and comprehensive training
Drawn a faculty strategic plan to better planning
Efforts to produce health professionals who are scientists as much as they are physicians
Enlistment of nearby hospitals for teaching purposes
Establish a small compensation for hospital service rendered by academic medical doctors

Establishment of a medical laboratory degree programme to train qualified technical individuals, some of whom can join college as support staff
Establishment of a premedical programme to prepare the students for medical programme
Establishment of a tripartite partnership (University –Ministry of Health-Community)
Establishment of collaborations (memorandum of understanding) with other hospitals in the country for clinical teaching purposes
Establishment of Education Development center for teacher training and capacity building for teachers, curriculum development, continuous professional development
Establishment of electronic libraries and improved educational resources
Establishment of Student-centered, community-based learning. The patient and the community are the focal points of instruction.
Exploring use of regional referral and district sites for teaching.
Faculty exchange from other medical schools in and outside the country to fill gaps
Focus upon principles of Primary Health Care within the curriculum
Gradual introduction of blended learning (a mixture of face-to-face supplemented by online materials)
Have experimented with extending the working hours of the academic staff in order to cover the practical programme but found that it was too hectic for the academic staff.
Implementation of new investment policies to increase medical school income
Improve and increase teaching resources – classrooms, laboratory space, library, computers, internet, equipments and apparatus
Improve teaching environment
Improved internet and computer facilities
Improvement of hospital facilities for clinical studies
Improvement of working environment
Improvements in administration
Incentives for staff
Increase lecturers' salaries at recruitment
Increase the number of medical doctors in our city to recruit clinical teaching staff and graduates
Increase the number of students at entry to school from 30 to 65
Increased intake despite inadequate facilities
Increased Laboratory Space
Increased library space.
Increased number of lecture theaters
Initiated workshops to fill training needs.
Initiation of Memoranda of Understanding (MoU) with the regional health bureau to use regional hospitals as training sites
Initiation of Memoranda of Understanding (MOU) to jointly appoint staff at secondary hospitals as teaching staff.
Inter-disciplinary training and emphasis on teamwork
Introduction of a Medical Education Department
Introduction of book bank that is offered to every student
Inviting Guests to fill faculty gaps

Library reorganization and increased access to books for students
Local training of our own staff
Making maximum use of available community settings
Master plan prepared to build a new faculty buildings and university hospital
Medical specialists teach specific topics
more funding
more staffing
more training hospitals and infrastructure
Negotiation with regional referral hospitals to offer better quality teaching sites.
Partner with other universities to accommodate students of BSc (Hons) Medical Science for clinical training
Procurement of land for building more lecture rooms to accommodate increase in intake.
Promotion of excellence in biomedical education by fostering library and information services
Putting a special focus on postgraduate training for basic sciences to train more teachers
Recruit more clinical teachers
Recruiting of new basic science lecturers
Regional hospitals used as training sites for Medical Doctors
Regulations allow repetition of courses and exams many times in each year.
Regulations allow students to carry on non-pre-requisite failures throughout the first three years.
Research and hands-on learning focused upon service to patients and community
Review staff recruitment policies and address negative areas
Seeking donor funding to supplement the University budget
Seeking more funds for universities from national income
Some students do not live on campus, to get round the problem of lack of accommodation
Stabilize the lecturers on duty in their position
Staff dedication
Student support
Training of instructors for basic science and clinical science (specialization and MSc training)
Training of more staff members
Training students in available Health services settings
Training Workshops for teachers
Transfer the supervision of the medical school from the Ministry of Education to the Ministry of Health
Use of donor funding to supplement faculty remuneration
Use of established nearby hospital(s) and staff as academic teaching hospital(s)
Use of modular curriculum to reduce failures due to numerous exams and to lessen the likelihood that a student loses a year in repetition.
Use of part time lecturers
Use of postgraduate residents in the various specialties to teach in the undergraduate programs as part of their training

Innovations Implemented to Address Barriers to Increasing Number and Quality of Doctors Trained: Central Africa
Access to dormitories free of charge for needy students
Connections to fiber-optic cables
Construction of new campuses
Construction of new library
Construction of new pedagogic complex
Cooperation with foreign schools to offer options in pharmacy and dentistry: Switzerland, France, Egypt, Sweden, etc…
Creating paths for specialists within priority areas
Development of cooperation with diversified resource implications human partner institutions in the activities of the institution
Development of Library
Digital Campus (Internet Access) development
Implementation of curricular changes including the Bachelors, Masters and Doctorate system, production of new courses syllabus, new tools for clinical postings
Implementation of training in Pedagogy of the Health Sciences for the Teachers
Inclusion of the Diaspora and their networks of relationships in their home countries in the activities of the institution
Infrastructure rehabilitation
Policy of human resource development through the promotion of teachers by their inclusion on the reserve lists of CAMES
Program evaluation
Rehabilitation and construction of new building through the PROACTP progamme
Rehabilitation of labs
Rehabilitation of locales
Set up new laboratories
The establishment of an office for student support
Use of an "Internal Purse"
Use of Formative Assessment (Continuous assessment)

Innovations Implemented to Address Barriers to Increasing Number and Quality of Doctors Trained: Southern Africa
Accelerated admission program for students from previously disadvantaged communities
Approaches for increasing throughput through a variety of student support strategies
Building modern teaching facilities
Construction of a state-of-the-art skill laboratory
Creation of teaching venues and equipment in peripheral hospitals
Development of a rural clinical school and community-based education approach.
Development of an excellent clinical skill laboratory.
Development of core curriculum based on burden of disease
Development of strong partnerships
Establishment of extended degree programme mainly at first year level to increase student numbers
Establishment of extensive student support systems
Establishment of new medical school(s)
Establishment of Practice of Medicine programme for early clinical exposure
Implementation of a Master’s programme in Health Sciences Education
Implementation of a rural students bursary scheme
Implementation of community-based education in hopes of bonding students with the rural population, thus encouraging more doctors to work in underserved areas
Implementation of mentoring and tutoring schemes for medical students
Implementation of recruitment and enrolment strategies which aim to address a legacy of educational inequality
Implemented an outreach programme to satellite sites used as sites to train registrars.
Implemented the Classroom Performance System: an electronic system used by lecturers to track class attendance, complete a weekly assessment and identify problems.
Implemented the Learning Development Programme; an academic support programme given to first year health sciences students who fail their first semester
Introduction of innovative admission procedures to increase access for rural students to medical education
Introduction of innovative learning methodologies into our medical training programme to improve the success rate of students.
Negotiation with provinces to transport students to various teaching sites in rural areas.
Recruitment of part time based staff to supplement full time staff

**Innovations Implemented to Address Barriers to Increasing Number and Quality of Doctors Trained: Western Africa**
Admit exchange medical students from other countries and vice - versa
Affiliate with donor agencies, such as the MacArthur Foundation
Affiliate with multi-centered Academic Hospitals to diversify clinical teaching opportunities and provide the immediate community with specialist expertise from the clinical faculty, who work as consultants at these academic health institutions
Aggressive recruitment with enhanced salary scales and positions, especially in semi-urban medical schools
All academic staff must possess the primary medical qualification of MBBS/MD as minimum to qualify to be on the faculty.
All students participate in a preparatory course and daily classes in Medical English for the duration of their studies. This will ease access to English scientific literature and allow participation in English-language lectures. All students are completely bilingual within their first year.
Application of the LMD (*Likely License, Masters, Doctorate*) system to facilitate student mobility
Batch students into manageable groups aiming to graduate several batches each year
Building of new and bigger lecture theatres
Clinical rotations are well organized, monitored and evaluated trimesterly
Collaboration with relevant stakeholders, MOH, University, Government to pay the cost of training for all medical students with the requirement that following graduation, they will then work for the country for 3 years with pay
Considering reducing tuition fees to assist students in financing their studies
Construction of internet facility
Construction of offices for staff
Construction of student hostels
Construction/renovation of teaching hospitals
Courting affiliations and international partners to remove barriers which are a tangible reality in our faculty
Creation of a private medical school to accommodate students who could not find places in public medical schools
Creation of pedagogical student groups by dividing large numbers into small groups which can meet in small rooms
Curricular hybridization; a combination of problem- based learning, community based initiatives and didactic traditional approaches for training
Curricular initiatives, including an Integrated System-based Medical Program
Decentered training centers in regional cells
Developed linkages with other Universities
Development of a Clinical Skills and Simulation Centre
Development of a medical education curriculum offering MD/PhD in addition to primary medical qualification, with the goal of producing MD/Scientists who will see academic medicine as a future career.

Development of networks of autonomous specialization in the core disciplines, clinical and public health and community health
Early introduction to the clinics
Employ part time teachers to reduce salary burden without decreasing the quality graduates
Encouraging individuals and organizations to donate buildings (wards, theaters, etc.) to the teaching hospital
Encouraging student participation in teaching and learning activities
Enhancing audiovisual teaching and learning support
Establish Post graduate medical training programmes to provide faculty for medical school
Establish Public-Private Partnerships to supplement government support to the University and the Medical School
Establish relationships with public hospitals to use the hospitals for clinical teaching
Establishing Public-private partnerships to develop hostels and provide services for students
Establishment of a Development Unit in the office of Vice-Chancellor whose major task is to identify people and organizations which can endow and invest in the University and the College.
Establishment of a Graduate Entry Medical Program
Establishment of a stringent, standardized entry system, using interviews for selection of final entry.
Establishment of new medical school(s)
Establishment of new medical school(s)
Expansion of physical facilities
Fund postgraduate program
Fund research
Funding training in modern day technologies for clinical lecturers in their specialties
Hire a faculty composed of high level professors from prestigious universities around the world
Hire more teachers.
Improvement of clinical rotations by increasing the number of slots and the supervision of rotations by teachers
Improvement of Facilities
Improvement of information technology
Improving the working conditions of the teachers and students
Increase in the number of admitted students for study
Increase in the number of employed teaching staff
Initiatives to improve internally-generated revenue, including operating a Clinical Diagnostic Centre and a Fitness Centre.
Invitation of lecturers from other institutions on sabbatical or visiting basis
Invite collaboration
Involve the state government and legislative body in collegiate activities where possible
Involvement in the development of Open Educational Resource material
Lobbying government to increase grant allocation
Movement of the Teaching hospital to a new site where facilities for clinical, radiological and laboratory teaching have been massively expanded and improved.
Multiplication of internship sites

Numerous clauses procedures at the end of first year reduce the percentage of student failure after the first year
Offering in-service training for lecturers in the basic science departments
Ongoing search for lecturers to occupy senior positions (Professors and Associate Professors) especially in the Basic Medical Sciences.
Operation of the Collegiate system in consonance with the National Universities Commission
Participation in student and staff exchanges
Partnership(s) to build university hospital(s) to increase enrollees and to supervise them effectively during hospital rotations.
Partnership(s) to set up skills labs (laboratoires de travaux pratiques) to increase enrollees and to supervise them effectively during hospital rotations.
Recruit young doctors into all departments in the Basic Medical Sciences. These are to undergo M.Sc and PhD programmes, thus raising a critical mass of medically-qualified teachers who are ideally suited to teach medical students.
Recruitment of more lecturers in the pre-clinical sciences
Recruitment of young teachers
Reform of programs with the support of the West African Health Organization
Regular Staff Recruitment
Scientific method and research are integral to entire course of study
Seek to raise capital and funding for the establishment of campus academic and social structures and practical training and research
Setting up a regular system of multiyear recruitment coupled with a premedical program to upgrade the capacities and abilities of students
Soliciting support from donor agencies
Strengthening of cooperation with the government of Cuba to support school activities
Teaching hospital facility improvement
Teaching hospital only appoints consultants who are academic staff in the college
The establishing of specialized laboratories
Training of Medical Doctors in their environment thus ensuring high productivity in the sub-region
Use of computer facilitated learning
Use of objectively structured clinical examinations
Work to make the college relevant in state activities
Work with the West African Postgraduate Medical College to strengthen Medical Faculty.
